# Reassessing boreal wildfire drivers enables high-resolution mapping of emissions for climate adaptation

**DOI:** 10.1126/sciadv.adw5226

**Published:** 2026-02-27

**Authors:** Johan A. Eckdahl, Lars Nieradzik, Louise Rütting

**Affiliations:** ^1^ Department of Earth and Environmental Sciences, Lund University, Lund, Sweden.; ^2^ Energy and Resources Group, University of California, Berkeley, CA, USA.; ^3^ Department of Earth Sciences, University of Gothenburg, Gothenburg, Sweden.; ^4^ Chair of Soil and Plant Systems, Brandenburg University of Technology, Cottbus-Senftenberg, Cottbus, Germany.

## Abstract

The expansive carbon reservoirs of the boreal region are becoming some of the most rapidly growing sources of greenhouse gasses under a positive feedback between intensifying fire activity and climate change. However, current regional-scale methods lack the spatial precision needed to improve understanding of the drivers of these fluxes to inform strategies aimed at maximizing landscape carbon storage. Here, we develop an alternative and highly constrained procedure for estimating wildfire emissions at both local (10 meters) and regional (1000 kilometers) scales in boreal Fennoscandia. This approach reassessed existing knowledge of heat development within the context of modern geospatial datasets, revealing expanded applications of satellite-derived fire radiative power in classifying distinct smoldering dynamics. The findings additionally emphasized the importance of capturing fine-scale variation in climate-sensitive fuel loading when determining regional fire season impact. Comparisons revealed substantial limitations in existing boreal carbon accounting methods while providing insights into the sensitivity of fire regime characteristics to climate, management, and landscape structure.

## Teaser

Fine-scale mapping improves regional carbon accounting.

## INTRODUCTION

The boreal region stores more carbon (C) than the atmosphere (*1*, *2*), largely in thick, flammable organic soil layers, and is one of the fastest growing sources of greenhouse gasses under more frequent and intense fires driven by climate change (*3*–*6*). This region is vital to the global land-atmosphere C balance, but comprehensive accounting of changing fire emissions has been limited by the sheer vastness and relative inaccessibility of northern landscapes (*7*). Current estimates rely heavily on remotely derived data, often using satellite metrics developed in lower-latitude ecosystems that lack sufficient ground validation under the unique fire dynamics of the boreal region (*8*–*12*). While improved calibration and additional constraints can, to some degree, reconcile the large emission variation found between major global biomass burning datasets, these methods still lack the spatial precision needed to allocate emission rates to boreal fuel configurations at the scale by which they can be protected and managed (*13*, *14*). Specifically, it is now difficult to quantify the influences of changing burn area extent, combustion completeness, and fuel structure on the measured increases in boreal wildfire emissions in recent fire seasons, limiting our ability to account for these factors when developing climate change adaptations aimed at maximizing landscape C storage (*6*, *15*). Therefore, improved mapping capabilities that connect wildfire emission rates to specific fuel dynamics at fine spatial scale are a key step forward in managing the resilience of boreal C stores under the coming decades of global change.

**Fig. 1.**
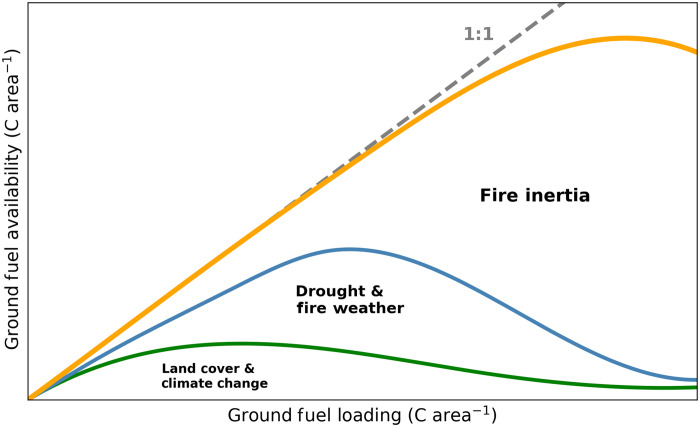
Conceptual model of how moisture dynamics at three temporal scales control forest floor fuel availability (i.e., maximum potential combustion rate). At the centurial scale, climate and site drainage set the baseline fuel load (*x* axis), while decadal shifts in moisture (e.g., land-cover change and climate variability) can modulate how much of that fuel is actually flammable (lower curve). Superimposed on these longer trends, seasonal drought and fire-weather conditions near ignition can sharply increase fuel availability (middle curve), enabling rapid fire spread in relatively dry and, therefore, lower ground fuel areas. A more instantaneous moisture dynamic is derived from heat release at the fire front as it spreads (fire inertia, upper curve), which can desiccate and volatilize adjacent unburned fuel loads, further priming them for combustion. These effects combine to produce a fire season–specific inflection point where an approximately linear relationship between fuel loading and fuel availability begins to drop off and eventually invert because of the remaining moisture limitations on fuel consumption. It is proposed that areas experiencing high-intensity burning have emission rates that tend to be more limited by intrinsic properties of their fuel loads (e.g., total amount), rather than instantaneous moisture conditions, due the priming effect by the fire front. Under this saturation of thermal energy, the complex interactions that determine fuel moisture conditions at the time of fire can be ignored, leaving the bulk of emission variation to be more simply modeled by the factors that influence longer-term fuel properties (e.g., climate and drainage).

**Fig. 2.**
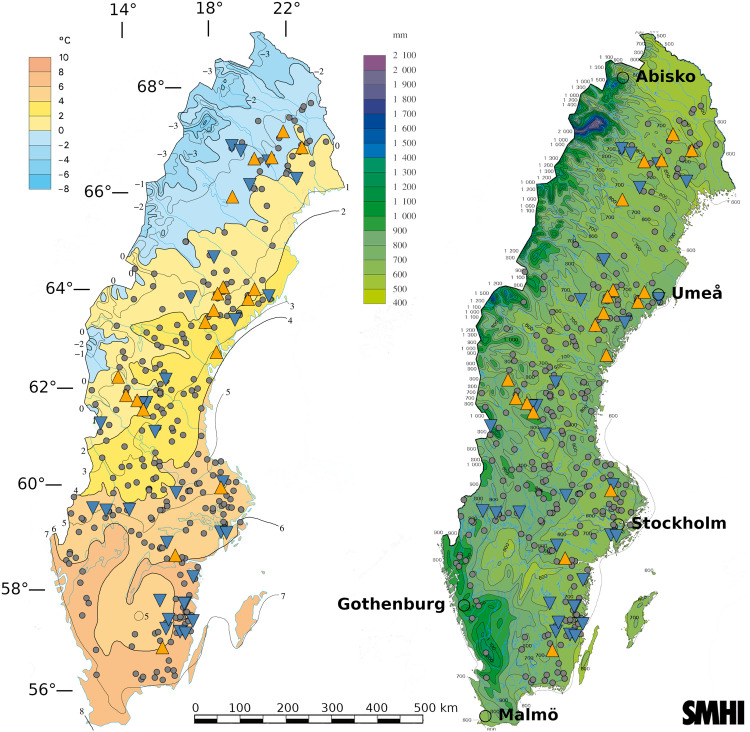
Climate maps of the study region. The 324 forest wildfires occurring during the 2018 fire season are shown as gray circles on a map of MAT (left) and MAP (right) over the normal period of 1961–1990 provided by the Swedish Meteorological and Hydrological Institute. The 19 high-intensity field–sampled fires are indicated by orange, upward-pointing triangles, while the 31 low-intensity field-sampled fires are indicated by blue, downward-facing triangles.

Here, we examine the drivers of boreal forest C emissions using our current understanding of the coupled heat development of aboveground and belowground wildfire combustion, aimed at bridging gaps between varied field- and remote-based observational approaches (Fig. 1). Data regarding commonly used fire weather parameters, satellite-derived burn intensity, and field measurements from a network of 50 regionally dispersed forest wildfire emission estimates in boreal Sweden (Fig. 2) were combined into a multiphasic modeling approach to provide an upscaled map of boreal forest C emissions for the region with a resolution of 10 m. This map was externally validated using previous field sampling and compared to six widely implemented biomass emission datasets to quantify the general importance of spatial precision in interpreting emission variation. A short discussion of the potential for improved understanding of the connection of landscape structure to emission dynamics using this mapping approach was also provided.

## RESULTS

### Satellites can identify secondary ignition of persistent smoldering belowground

Fire weather has long been considered a primary driver of boreal wildfire emissions due its role in desiccating fuel loads and producing dry winds, together facilitating intense fire spread (*16*, *17*). In lower-latitude systems, fire radiative power (FRP), a direct measurement of burn intensity, has been shown to correlate with the instantaneous rate of aboveground combustion of organic material (*8*, *9*). However, despite common usage, this relationship remains poorly validated in the boreal region, where most emissions originate from belowground soil organic layer carbon (SOLC) (*18*–*20*). Moreover, recent large-scale analyses of extreme wildfire events across boreal North America have found that variation in fire weather-induced drying has limited influence on C emissions when considered alongside field measurements of fuel loading (*18*), although the upscaling of field measurements has remained weakly resolved because of limited ground fuel load mapping and little explanation for variation in observed differences in strength of the fuel load to emission relationship (*7*, *18*). While at first glance these conflicting observations might appear to inhibit the ability to place constraints on boreal wildfire emissions, we argue that they are complementary to that end when the distinct development stages of wildfire are carefully considered.

Of our 50 burned field plots, MODIS (MODerate resolution Imaging Spectroradiometer)-derived FRP signals were detected in 19. Signal absence, despite intense fire activity, can reflect obstructive smoke or cloud cover, small burn areas, missed overpasses, or algorithmic rejection (*21*). Nonetheless, logistic regression incorporating the variables in Table 1 determined the occurrence of an FRP signal with 88% accuracy (fig. S5A) using final fire size (BurnArea), wind speed during ignition (WindSpeed), stand overstory biomass (OverstoryBM), and site drainage [topo-edaphically derived moisture (TEM)]. These results indicate that FRP effectively signaled a threshold of weather- and landscape-controlled intensity of fire development.

**Table 1.** Independent variables used in regression model selection. Fire weather parameters and Canadian Fire Weather Index values were taken on date of burn.

Category	Variable	Unit	Description
Climate	MAT	°C	Average mean annual temperature over 1961–2017
	MAP	mm	Average mean annual precipitation over 1961–2017
Stand properties	TEM	–	Topo-edaphically derived moisture conditions
	OverstoryBM	tonnes ha^−1^	Remotely derived overstory biomass
	StandAge	year	Stand age at time of fire
Satellite fire measurements	FRP	MW	Fire radiative power derived from MODIS
	dNBR	–	Differenced normalized burn ratio derived from Sentinel-2
	BurnArea	–	Natural logarithm of final burn scar area from Sentinel-2
Fire weather parameters	DOB	–	First reported Julian date of burn
	Temp	°C	Air temperature at 14:00
	WindSpeed	m s^−1^	Speed of wind at 14:00
	RH	–	Relative humidity at 14:00
	Prec	mm	Cumulative precipitation at 20:00
	HBV	–	Modeled instantaneous soil moisture balance
Canadian Fire Weather Index	FFMC	–	Fine Fuel Moisture Code
	DMC	–	Duff Moisture Code
	DC	–	Drought Code
	ISI	–	Initial Spread Index
	BUI	–	Buildup Index
	FWI	–	Fire Weather Index

Furthermore, plots with detected FRP signals exhibited greater vegetation destruction, as indicated by a significantly increased mean differenced normalized burn ratio (dNBR; 464.3 versus 353.9) (*11*, *12*), and a shifted control over SOLC emissions from fire weather to ground fuel structure. This shift was evident in regression model selection using fire weather variables in Table 1 alongside prefire SOLC: In FRP sites, SOLC alone explained emission rates [coefficient of determination (*R*^2^) = 0.748, fig. S5B], whereas, in non-FRP sites, emissions were influenced (*R*^2^ = 0.431, fig. S5C) by SOLC (β = 0.656) and two fire weather latent variables (β = 0.857 and β = −0.313). In addition, FRP sites released over eight times more SOLC on average (1.65 versus 0.19 kg C m^−2^, *P* = 0.044), with a stronger coupling between emissions and fuel loading in this compartment (bivariate *R*^2^ of 0.748 versus 0.255, *P* = 0.004). The magnitude of the FRP signal, however, did not directly correlate with SOLC loss (*P* = 0.453, *n* = 19), nor did the magnitude of dNBR in FRP (*P* = 0.309, *n* = 19), non-FRP (*P* = 0.788, *n* = 31), or all sites (*P* = 0.171, *n* = 50).

These results suggest that in boreal ecosystems satellite-derived observations can be more valuable for mapping transitions in fire dynamics that are responsible for handing over SOLC emission controls from fire weather to intrinsic fuel structure, rather than directly determining C emission rates. Once initiated by sufficient energy deposition from intense aboveground burning, the soil smoldering feedback cycle is sustained in place by internal heat generation that exceeds dissipation, persisting for weeks to years under occluded remote visibility, resisting even large inputs of moisture or thick seasonal snow cover (*22*–*24*). This environmental buffering was evidenced by the release of SOLC emissions from short-term moisture control alongside an enhanced coupling to fuel loading, suggesting that FRP can serve as a reliable indicator of degrees of aboveground burn intensity capable of stimulating a secondary ignition of persistent smoldering combustion belowground.

Temporal and spatial undersampling of weather, moisture conditions, and fire energetics near the time of burn has been considered a major barrier to estimation of C emissions across the boreal region (*25*, *26*). However, the robust statistical relationships identified here suggest that determining areal emission rates from high-intensity wildfires are more limited by the accuracy and spatial coverage of factors influencing the longer-term prefire conditioning of SOLC. Therefore, further investigation of ground fuel properties that determine its combustibility under the instantiation of the smoldering combustion phase, especially those that are climate sensitive, is key for spatially fine-scale predictions of boreal wildfire emission rates under future fire and climate regimes.

### Climate and drainage data provide finely resolved ground-fire emission maps

Previous study of the 50 field sites found the SOLC to emission relationship to be further constrained when including information regarding the organic layer carbon-to-nitrogen (C:N) ratio and the amount of char produced during burning (*19*). The C:N ratio reflects detrital input chemistry and its decomposition state, affecting heat release during oxidation (*27*, *28*). Char production is linked to time-extended combustion efficiency (*29*–*31*) and, in our plots, was responsible for mechanical densification of the fuel surface (*19*), potentially determining smoldering duration by providing for its eventual suffocation. These three fuel parameters (i.e., SOLC, C:N, and char rate) were in turn controlled by long-term-averaged mean annual temperature (MAT), mean annual precipitation (MAP), and TEM (a metric of soil drainage) (*19*). Fire dynamics that link fuel properties to emission rates would then also enhance the coupling of emissions to climate and drainage. Therefore, in the absence of suitable fuel property maps for the region (section S4), climate and drainage would be important factors for the mapping of SOLC emissions.

In high-intensity field plots (FRP signal presence, *n* = 19), MAT (β = 0.592), MAP (β = 0.875), and TEM (β = 0.256) strongly explained SOLC emissions (*R*^2^ = 0.695) using the equation

$$C_{\text{loss}}=0.5121\cdot \text{MAT}+0.0328\cdot \text{MAP}+0.0173\cdot \text{TEM}-23.2920$$ (1)

However, MAT, MAP, and TEM together did not significantly explain SOLC emissions in low-intensity fire (no FRP signal; mean: 0.191 ± 0.317 kg C m^−2^) nor did they significantly explain variation in understory emissions in high (mean: 0.081 ± 0.011 kg C m^−2^)– and low (mean: 0.066 ± 0.008 kg C m^−2^)–intensity fire. This is likely due to prefire understory C having no measured controls alongside near complete removal during fire, leaving little burn variation to sample (*19*). In addition, low-intensity SOLC emissions were instead more strongly controlled by transient and difficult-to-predict fire weather conditions as described above. Combustion from additional C storage pools, including forest canopy, is typically considered negligible across the region’s mostly fire-resisting vegetation (*20*), and virtually no canopy blackening was found across the 50 field sites (*19*). Therefore, spatially explicit rasters of MAT, MAP, and TEM were combined in Eq. 1 to provide a 10-m resolution map of SOLC emissions only in areas burned under high intensity (i.e., MODIS FRP signal present) within the 324 fires occurring during 2018. Average values for the remaining three C emission sources were otherwise applied at 10-m resolution to their respective burn conditions. It was found that organic soils burning under high intensity released 95.9% of an estimated 0.507 ± 0.080 Tg C total. This means that most of emission variation for the fire season was captured under high statistical constraint by the variables MAT, MAP, and TEM.

Hydroclimatic factors impose fundamental limits on the flow of radiative, thermal, and chemical energy in ecosystems. While biotic interactions modulate C storage within these boundaries (*32*, *33*), our findings show that climate and drainage alone can exert strong direct constraints on wildfire emission rates in areas where fire weather and landscape structure promote sufficient combustion energy for the ignition of soil smoldering. With simple time- and space-specific field calibration (section S3), our modeling approach for SOLC emissions may extend to the large areas of *Pinus sylvestris* forests of western boreal Eurasia and even fire-resisting boreal vegetation in eastern Eurasia and North America, where heat primarily develops close to the ground (*19*, *34*).

However, regions dominated by canopy fire that generates heat at a distance from the soil may exhibit different thresholds for triggering belowground smoldering combustion, with the effects of climate on emission rates exhibiting additional mediation by variation in aboveground fuel structure (*18*, *34*). Despite this added complexity, site drainage has been an important factor for determining wildfire emissions at local scales (10 m) across a variety of boreal forest types (*18*, *35*). The importance of climatic variation as we have shown, however, has largely been overlooked, likely due to low regional spread and burn scar replication of individual field studies, presenting an important opening for further investigation (*19*). Despite belowground C typically dominating emissions in boreal wildfires, it remains poorly statistically constrained (*7*, *10*, *18*, *35*). Incorporating spatially fine-scale climatic and hydrological analysis alongside multiphasic modeling of fire dynamics, as demonstrated here, can improve spatial accuracy of wildfire emission estimates across the boreal region as a whole.

### Seasonal emissions are highly sensitive to fine-scale, location-specific fuel loading

**Fig. 3.**
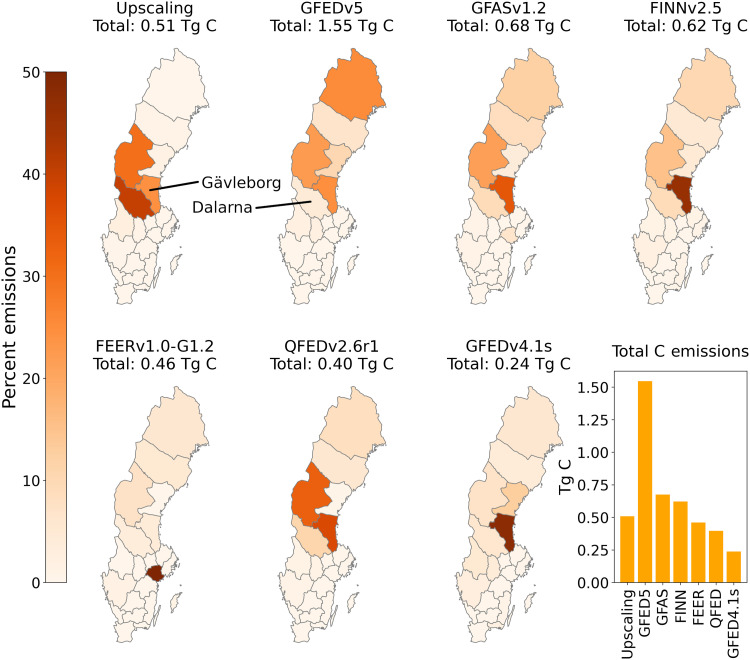
Comparison of upscaling method to six major global biomass burning datasets. Large discrepancies in total wildfire C emission estimates are revealed when comparing available emission data for Sweden during 2018. While the global models demonstrated sporadic spatial allocation of emissions, they tended to overestimate emissions in the county of Gävleborg, which had large fire sizes (average 327.2 ha), but relatively small areal emissions due to low ground fuel loading (1.3 kg C m^−2^). In contrast, emissions tended to be underestimated in the county of Dalarna, which had smaller fires (average, 154.2 ha) but much higher areal emission rates due to increased ground fuel loading (5.08 kg C m^−2^). While these six models may demonstrate greater consistency at a global scale, they do not appear to perform at their native resolution (i.e., 0.1° to 0.25°) because of insensitivity to variation in landscape fuel loading.

The emission values derived from our upscaling procedure (table S1 and fig. S4) were compared against six global fire emission datasets: the Global Fire Emissions Database (GFEDv5 and GFEDv4.1s), Global Fire Assimilation System (GFASv1.2), Fire INventory from NCAR (FINNv2.5), Fire Energetics and Emissions Research (FEERv1.0-G1.2), and Quick Fire Emissions Dataset (QFEDv2.6r1), at the scale of Nomenclature of Territorial Units for Statistics (NUTS) 3 regions (administrative county, Fig. 3). National emission rates from these datasets ranged from approximately half to three times (1.05 to 6.75 kg C m^−2^) that calculated through our field-based upscaling for the 229 km^2^ burned in Sweden during summer 2018 (2.22 ± 0.35 kg C m^−2^). The six datasets exhibited scattered spatial distribution of emissions across counties but tended to overestimate emissions from the large, intense fires with shallow ground fuel layers in Gävleborg county (upscaled estimate: 1.30 kg C m^−2^ over 94.90 km^2^, 327.2 ha average fire size) while underestimating the nearly quadrupled emission rates from the deep soil organic soils in the smaller fires of Dalarna (5.08 kg C m^−2^, 40.09 km^2^, 154.2 ha).

To externally validate our methodology against a separate fire season and intraburn scar field estimation, we applied our 10-m emission map to the 420 equally spaced field plots from previous study of the Sala megafire occurring in central Sweden during 2014 (*20*). The upscaled emission rate of 3.79 kg C m^−2^ aligned closely (15.7%) with the 4.5 kg C m^−2^ estimated in the field. However, the six global datasets estimated emissions at only 1.16 to 2.66 kg C m^−2^. The Sala fire spanned 131 km^2^ of diverse fuel structure, including shallow upland forest soils and deep peatlands, with drained peatland areas in particular emitting up to 15.6 kg C m^−2^ (*20*). More precise calibration of emission estimates to the specific conditions of the Sala fire, particularly accounting for human-induced artificial drainage not fully incorporated into TEM, could have provided even greater alignment with field measurement. Nonetheless, our higher-level approach, which relies on climatological normals and topo-edaphic factors, yielded estimates sufficiently consistent to support interseasonal comparison (section S3). Notably, upscaled emission estimates across the entire Sala burn area reached 0.487 Tg C. This single fire complex, which comprised all notable wildfire activity in Sweden during 2014, produced C emissions equivalent to 96% of the total estimated emissions from 324 fires and nearly double 229 km^2^ of area burned during the 2018 fire season.

While the six emission datasets may provide greater consistency at a global scale (*36*), our findings reveal that they were not suitable for capturing the complexity of boreal fuel loading and fire propagation at their native resolution (i.e., 0.1° to 0.25°) due to insensitivity to variation in landscape ground fuel loading. These limitations not only obscure critical assessments of land-atmosphere C fluxes but also hinder our ability to identify the drivers behind varied emission rates. For example, the much greater burned area and regional dispersion of fire events in Sweden during 2018, compared to 2014, might be mistakenly attributed to a sharp climate change-driven transition toward higher emitting fire seasons when relying on these remote datasets alone. However, our field-based estimates show that the two seasons had roughly equivalent C emissions due to the chance propagation of fire over a relatively small area of highly emitting fuel structure during 2014.

Although the greenhouse gas emissions from Sweden’s burned area in 2018 might be considered minor compared to the recent extreme fire seasons in Siberia and Canada (*14*, *37*), similar lack of discernment in these events is likely to go unnoticed in the absence of integrated multimodel and field-based comparisons (*3*, *14*). Even if better constraints can be placed on total regional emission estimates, without improved insight into spatial variation of changing emission drivers, attributing seasonal variation in fire emissions to factors such as spread of burning into deeper fuel loads or to intensifying fire dynamics that increase combustion completeness would remain limited. This complicates decision-making such as prioritizing protection of dense C reserves in traditionally fire-avoiding ecosystems or mitigation of fire intensity in more frequently burned areas (*15*, *38*–*40*). Accurate, fine-scale mapping of belowground fuel structure and associated emission rates is therefore essential for both quantifying fire impacts on greenhouse gas budgets and informing effective forest management strategies.

## DISCUSSION

Previous regional assessments of boreal wildfire have faced substantial limitations in both the precision and spatial scale of estimating ground C emissions (*10*, *14*). In contrast, the current study of the 2018 fire season in Sweden introduced highly constrained methods capable of estimating emission rates at both local (intrafire, 10 m) and regional (interfire, 1 to 1000 km) scales. The multistage modeling approach emphasized the critical role of variation in fire weather in amplifying seasonal emissions by promoting high-intensity fire fronts. But while high-intensity fire did dominate most of the 2018 burn area, 77% of individual fire events burned with less than half their area under these conditions. In addition to the controls of fire weather, strong negative correlations were observed between county population density and both fire size and intensity (section S5), revealing a potential influence of anthropogenic factors such as forest fragmentation and rapid fire suppression (*15*, *41*–*43*). Without these interventions, the spatial extent of high-intensity burns—and consequently, seasonal C emissions—would likely have been much greater. However, despite the importance of fire weather in shaping fire behavior, it provided little direct explanation for total emission rates across the region’s total burn area, which were instead governed by climate- and drainage-controlled variations in ground fuel loading. This divergence in explanatory factors highlights two potentially competing perspectives: Land managers may prioritize fire weather as the key driver of emissions with intention of minimizing regional high-intensity fire activity (*16*, *44*), while researchers focused on understanding that extreme burn complexes may emphasize the importance of fuel characteristics within their selected study areas (*18*, *35*, *39*). Our findings demonstrate that these perspectives are complementary aspects of a broader understanding of fire activity. Their integration is essential for improving the prediction and modeling of boreal wildfire C emissions in ways that are both practical for climate change adaptation and suitable for making interseasonal comparison of shifting fire regimes.

The need for more holistic analysis of fire season activity becomes increasingly evident when comparing high-resolution fire mapping to available forest geospatial data. Our observations of intense fire development in low-biomass areas with high wind speeds discussed above, coupled with disproportionate fire occurrence in young forests (section S5), motivate a hypothesis that clear-cutting practices facilitated uncontrollable fire spread during the 2018 fire season. Artificially abrupt transitions from areas of intense fire development can provide the energy needed to overcome the fire resistance of large C stores in older forests and wetlands, potentially explaining the observed disproportionate burning of high-biomass and poorly drained areas (section S5) (*41*). The Sala fire showed how fire spreading into drained peatlands drives catastrophic C losses, which can be severely underestimated by remote methods. Granular, ground-truthed emission mapping can reveal and address these deficiencies in accuracy and spatial allocation of C release while also providing insights into the role of landscape pyrodiversity in enhancing fire resilience (*45*, *46*). For instance, low-severity burn areas can buffer the impacts of extreme fire in adjacent areas by supplying reproductive material and nutrients that stimulate regrowth (*20*, *47*). Rapid recovery of primary production is critical for reducing postfire net C emissions that are capable of reaching those of active burning within only a few years (*48**–**50*). Further research into land management strategies, such as rewetting drained areas and maintaining ecosystem structure that dissipates fire energy around critical areas, is essential for safeguarding the C stores of boreal landscapes under intensifying fire regimes (*51**–**56*).

The rapid rate of climate change challenges these goals. Strong equilibration of C loading and emission rates with MAT and MAP in our observations offers insight into future fuel structure, granting an ability to identify emission hotspots long before they burn. However, the potential for persistent ecosystem reorganization under climatic disequilibrium will require continuous monitoring of all fire-sensitive C pools to ensure accurate model calibration and bias corrections (section S6). While substantial progress has revealed the vital role of boreal C stores in global climate regulation, further effort remains critical to inform strategies for protecting this important environmental service. Our study demonstrates the value of reconciling varied research perspectives under continued methodological development to provide more effective understanding of ecosystem C storage aimed at adapting to a changing boreal landscape.

## MATERIALS AND METHODS

The current study used 50 forest wildfires that were part of a field survey of the summer 2018 wildfire season in Sweden (*19*, *47*, *48*, *57*, *58*). Additional satellite and fire weather data were acquired to upscale field measurements to the entire 2018 burned area for Sweden. This upscaling methodology was compared to C emission estimates for the region from six global biomass burning models. Publicly available geospatial data regarding forest structure and human population density were further analyzed for their connection to emission rates across the region.

### Field site selection and data collection

Because field site selection and sampling methodology has been thoroughly covered in previous publication, information relevant to the current publication is provided in section S1, with additional information found in (*19*). In brief, the 50 burned plots were selected from a pool of all 324 mapped burn scars above 0.5 ha, which occurred in Swedish forests during 2018, with aim to maximize their span across gradients of MAT and MAP (Fig. 2). MAT and MAP values were averaged over the period 1961 to 2017 with intent to capture a time-integrated characterization of the temperature and precipitation patterns of each plot, with the averaging period motivated in section S3. Each burned field site was matched using a series of strict filters with an adjacent unburned control, which represented prefire conditions. The most important feature for matching ground C stocks was a 10-m resolution map of topo-edaphic moisture potential (TEM) provided by the Swedish Environmental Protection Agency (*59*) as integer values ranging from 0 to 240 (in order of increasing moisture potential). These measures gave an estimate of soil drainage, which can explain long-term soil moisture patterns conducive of ground C storage (*7*, *19*, *35*). Two additional data sources for matching were stand age (StandAge) and overstory biomass (OverstoryBM), which were provided as spatially explicit 12.5-m resolution raster files for the country of Sweden (*60*). Emission estimates were derived by subtracting field-measured burned plot C from their associated control plots at 1 year postfire. Little to no canopy blackening was found across the 50 plots, and, therefore, all C emissions were assumed to come entirely from soil organic layer and understory plant stores (*19*).

### Burn date, fire weather, and instantaneous moisture balance data acquisition

Data regarding burn dates used for the 50 burned field sites were provided by the Swedish Civil Defence and Resilience Agency (Myndigheten för civilt försvar, MCF). These dates were determined by the initial time of report of fire activity to local emergency services, spanning from 15 May to 3 August 2018. Date of burn (DOB) was used as a Julian date (integer from 1 to 365) in analysis. DOB is commonly used as the date on which weather parameters are extracted for analysis. It is also often used directly as an explanatory variable of fire emissions under the expectation that forests experiencing ignition times later in the year will have had more time to dry under continuous drought conditions, such as occurring throughout Sweden during 2018 until the end of the fire season (*61*), and thus become more flammable (*18*). However, DOB does not explicitly acknowledge features of the ecosystem such as typical snowmelt timing, fluctuating patterns of temperature and precipitation, nor fuel structure that control rates of desiccation.

MCF also provided fire weather and instantaneous soil moisture data at 11-km resolution at daily intervals throughout the fire season. These data were extracted from the weather model MESAN (Mesoscale Analysis System) developed by Swedish Meteorological and Hydrological Institute (SMHI). Fire weather included air temperature (Temp), relative humidity (RH), and wind speed (WindSpeed) at 14:00 local time. Daily precipitation (Prec) is given at 20:00 local time. Over the fire season, weather variables were used to calculate values for the Canadian Fire Weather Index (FWI) system (*44*). The FWI system consists of several variables that account for the effects of fire weather and instantaneous fuel moisture on fire behavior. The FWI system variables used in analysis were the Fine Fuel Moisture Code (FFMC), Duff Moisture Code (DMC), Drought Code (DC), Initial Spread Index (ISI), Buildup Index (BUI), and the highest level Fire Weather Index (FWI). The variable HBV describes instantaneous moisture in the duff layer, similar to the FWI system’s parameters DMC and DC, which have only calculations based on weather conditions. The HBV value is calculated using weather conditions but additionally integrates the soil moisture holding capacity of organic soil layers (*62*). The HBV value was extracted and provided by MCF from the larger HBV hydrology model developed by SMHI. The most proximal value for all MCF-derived variables on the burn date was assigned to each of the 50 wildfire field plots for use in all statistical analysis. Further detail on how these variables were expected to influence fire behavior is given in section S2.

While fire weather variables, such as DOB, DC, and HBV, have specific formulations that carry unique statistical information, their similarities produced issues with multicollinearity in regression. When used in multiple regression, fire weather variables were first transformed into the five most explanatory latent variables for a given dependent variable using partial least-squares regression (PLS). PLS was performed using the class PLSRegression from the Python package scikit-learn PLSRegression from the Python3 package scikit-learn (*63*).

Previous analyses of the study plots found that drought indices and season-specific weather variables, such as the Standardized Precipitation Evapotranspiration Index, temperature, and precipitation anomalies, were not found to contribute significantly to emission variation (*19*). While incorporating high-temporal-resolution temperature and moisture interaction with specific vegetation types at individual burnt stands under more detailed mechanistic modeling may improve explanation of fuel loading and combustion availability during the fire season, such data are complicated to produce, especially if requiring intensive and spatially explicit monitoring of ecosystem structure. In contrast, we emphasize easier-to-acquire and commonly used fire parameters and climate variables averaged over the climatological normal period to enable interseasonal comparisons within a consistent, higher-level conceptual development.

### Satellite data acquisition

MODIS data were produced by two satellites (Terra and Aqua) that provided up to two FRP data points over each of the field plots. Both satellites use an identical sensing instrument that can measure the intensity of thermal radiation emitted by wildfires that reach each of its pixels (500-m ground resolution). This signal is converted to FRP values in units of megawatts (*64*). FRP values are, therefore, affected by the spatial distribution of thermal radiation produced within each pixel coverage area, which, in turn, can be occluded by, for example, forest canopy, smoke, or cloud cover on the way to the satellite (*64*). FRP data for the year 2018 were extracted from the Fire Energetics and Emissions Research webpage (*65*). The nearest FRP value within 1 km was used for each burned plot. FRP dates all corresponded with fire dates recorded by MCF. An attempt was made to integrate FRP values within 1 km of each field site over time to produce the metric fire radiative energy. However, this was not possible for enough field sites for use in statistical analysis likely due to a combination of poor satellite temporal resolution, fire front speed, and more time-extended burning tending to occur belowground. Throughout the text, the term “fire intensity” refers to the instantaneous rate of thermal radiant energy released due to burning per two-dimensional area. In the case of FRP, this would be radiant energy per earth surface area averaged within a MODIS pixel.

For the years 2017 and 2019, cloud-free L2A bottom-of-the-atmosphere Sentinel-2 images were collected for each of the 50 plots. The images were downloaded from the Copernicus Open Access Hub. Most images were taken mid-July, but some were taken during June or August when cloud-free images were, otherwise, not available for July. NBR was calculated from Sentinel-2 bands 8 and 12 using the equation

$$\text{NBR}=\frac{B8-B12}{B8+B12}$$ (2)

dNBR was calculated by subtracting the 2019 dNBR value from the 2017 value and multiplying by 1000, resulting in higher values indicating more severe burning.

### Data analysis and model construction

This study used hypothesis testing methods, correlation analysis, simple regression, multiple regression, and geospatial analysis within the Python 3 interpreter and the graphical user interface of the QGIS software environment (*66*). For all analyses, unstated *P* values are below 0.001. Explanatory variables using in analysis are listed in Table 1.

#### Significance testing

The nonparametic Mann-Whitney *U* test was used to test for significance differences (*P* < 0.05) between variable distributions. This test was used because of relatively small and varied sample sizes and poor accordance with parametric assumptions of some of the data distributions. The test was performed using the stats.mannwhitneyu function from SciPy in Python 3 (*67*).

#### Correlation analysis, simple regression, and multiple regression model selection

All regression analyses used the ordinary least-squares approach to estimate a function for a single response variable based on linear combinations of the predictor variables and an intercept term. Simple regression was performed using the stats.linregress function from SciPy in Python 3 providing significance (*P*), Pearson’s correlation coefficient (*r*), the coefficient of determination (*R*^2^), and the regression equation.

Multiple regression was carried out with the OLS class in the Python 3 statmodels package (*68*). Outputs included *P* values, the coefficient of determination (*R*^2^), standardized regression coefficients (β), Akaike and Bayesian information criterion (AIC and BIC), and the regression equation in original units. Forward model selection proceeded by beginning with the dependent variable producing the highest $R_{\text{adj}}^{2}$ value and adding the variables, one at a time, that most increased this value. Corrected AIC (AICc) was calculated as

$$\text{AICc}=\text{AIC}+\frac{2k^{2}+2k}{n-k-1}$$ (3)

where *k* is the number of model parameters and *n* is the sample size (*69*). ΔAICc and ΔBIC values were produced from the difference of the current model from the lowest AICc and BIC in the entire model selection process, respectively. ΔAICc and ΔBIC are two commonly used metrics to reduce model overfitting when adding model parameters (*69*). ΔBIC tends to select more parsimonious models than ΔAICc (*69*).

#### Logistic regression

Logistic regression was performed using the Logit class in the Python 3 statsmodels package. This approach used the maximum likelihood estimation method to model the log-odds of a binary outcome variable as a linear combination of one or more predictor variables. Key outputs from this analysis included *P* values for the significance of each predictor and various measures of model fit, such as pseudo *R*-squared ($R_{\text{pseudo}}^{2}$). Forward model selection proceeded the same as in multiple regression except using $R_{\text{pseudo}}^{2}$ instead of $R_{\text{adj}}^{2}$. However, because $R_{\text{pseudo}}^{2}$ is not an absolute metric like the coefficient of determination in regression, the average prediction accuracy of leave-one-out cross validation was used to determine model performance.

#### C emission upscaling procedure and error analysis

The procedure for upscaling emission estimates is described here. First, the 324 fire perimeters used during site selection were divided into high-intensity burned areas as indicated by being with 1 km of a MODIS FRP signal. The remaining burned area was classified as low intensity. A multiple regression equation was produced using MAT, MAP, and TEM values to explain organic layer C loss in the 19 field plots that were classified as having high-intensity burning. Rasters for these variables were combined in QGIS in accordance with the regression equation to provide a map with 10-m pixel size (matching TEM raster resolution) of emission rates from organic soils burned under high intensity. Because MAT, MAP, and TEM did not suitably explain variation of C emissions from organic soils and understory vegetation burned under low intensity or understory burned under high intensity, average field-derived values were applied to these emission sources on the 10-m resolution map.

To calculate error in regression analysis the root mean square deviation (RMSD) was calculated as

$$\text{RMSD}=\sqrt{\frac{1}{n}\sum_{n=1}^{N}{\left({\hat{y}}_{n}-y_{n}\right)}^{2}}$$ (4)

where *n* is the number of variables in a regression and ${\hat{y}}_{n}$ is the regression prediction of the actual *n*th dependent variable *y_n_*.

Error for mean values of variable distributions was calculated using the SEM

$$\text{SEM}=\frac{\sigma }{\sqrt{n}}$$ (5)

where σ is the SD of the variable and *n* is the number of values in the variable distribution.

Both the RMSD and SEM error values are indicated by the ± symbol. RMSD was applied to each pixel in the 10-m emission map that was calculated using regression (i.e., high-intensity organic layer C emissions). SEM was applied to all other emission sources (i.e., low-intensity organic layer and understory as well as high-intensity understory C emissions).

#### Geospatial analysis

All raster data analysis was performed in the QGIS graphical interface or the Python 3 interpreter. Field plots were represented in QGIS by a 10-m radius circle. Using the Zonal Statistics tool, pixel values for rasters of MAT, MAP, TEM, OverstoryBM, and StandAge extracted from (i) within Sweden as a whole, (ii) within the 324 burn scar boundaries, and (iii) averaged within each 10-m radius circle centered on the 50 burned plots.

For more interpretable tabulation and charting, the values of the current study’s generated 10-m C emission map were conglomerated within county polygons outlining the level 3 areas (i.e., administrative county) determined by the Nomenclature of Territorial Units for Statistics (NUTS). These values were separately conglomerated into NUTS level 2, NUTS level 1, and the country of Sweden as a whole. This procedure provided output data that captured emission variation ranging from 10 m to the large 1000-km scale at which the 2018 fire season occurred in Sweden.

### External validation and comparison

Field emission estimates from the largest wildfire in Sweden during 2014 (here, called the Sala fire) were provided by (*20*) and compared to the current study. Sampling of the Sala fire comprised 420 equally spaced plots within the burn scar. Emission estimates from peaty soils used the adventitious root method (*70*). Otherwise, soil emission estimates used control plots surrounding the burn scar to estimate prefire C. Understory loss was assessed and included in C emission estimates, but overstory and large wood on top of the forest floor were not included because they were deemed to contribute minimally (*20*). The current study’s 10-m resolution C emission map was also applied to each of the 420 field sites as well as within the entire perimeter of the 2014 Sala fire (*20*), providing for external validation of the upscaling methodology at the burn scar scale.

Daily values in 2018 for total C emissions from the Global Fire Emissions Database (GFEDv5 and GFEDv4.1s), Global Fire Assimilation System (GFASv1.2), Fire INventory (FINNv2.5), Fire Energetics and Emissions Research (FEERv1.0-G1.2), and Quick Fire Emissions Dataset (QFEDv2.6r1) were downloaded from their respective webpages. Because QFEDv2.6r1 and FINNv2.5 did not have results for total C emissions, their values for CO_2_ (carbon dioxide), CO (carbon monoxide), and CH_4_ (methane) were summed, representing the large majority of C emissions as indicated by the emission factors used in these models. For each of the six models, their daily C emissions were summed over the months of May, June, July, and August 2018 within the land area of each of the administrative counties in Sweden in the QGIS software environment and used for comparison to the upscaled field measurements produced by the current study.

## Supplementary Materials

### This PDF file includes:

Supplemental Texts S1 to S6 Figs. S1 to S5 Table S1 References
