# Supplementary material for: Reassessing boreal wildfire drivers enables high-resolution mapping of emissions for climate adaptation
Source: Sci Adv. 2026 Feb 27;12(9):eadw5226. doi: 10.1126/sciadv.adw5226 (PMC12947878; doi:10.1126/sciadv.adw5226)
Supplement: Supplementary file 1 — Supplemental Texts S1 to S6 Figs. S1 to S5 Table S1 References [file sciadv.adw5226_sm.pdf]

Science Advances
AAAS

Supplementary Materials for

Reassessing boreal wildfire drivers enables high-resolution mapping of emissions for climate adaptation

Johan A. Eckdahl *et al.*

Corresponding author: Johan A. Eckdahl, johan.eckdahl@berkeley.edu

Sci. Adv. **12**, eadw5226 (2026)
DOI: 10.1126/sciadv.adw5226

This PDF file includes:

Supplemental Texts S1 to S6
Figs. S1 to S5
Table S1
References

S1 Additional information regarding field site selection, data collection and previous data analysis

For the 50 wildfire field sites, site selection, field sampling and data analysis methodology used for estimating C emissions were covered in previous publication and therefore only briefly described here (*19*). The 50 wildfire network was designed to determine the role of climate in controlling boreal wildfire emissions and subsequent forest recovery. The field site selection therefore proceeded through a series of filters to meet this end.

Site selection began with a map of all burn scars greater than 0.5 ha (324 in total) identified during the summer 2018 period by the Swedish Forest Agency. Perimeters were drawn around the burn scars using the normalized burn ratio (NBR) values derived from Sentinel-2 bottom-of-atmosphere corrected bands 8 and 12. Each of the 50 selected field sites consisted of a 20×20 m^2 plot located within distinct burn scars (greater than 2 km separation) to reduce potential for pseudoreplication or spatial autocorrelation (*71*). Plot-wide values for raster data were taken as the average pixel value within a 20 m diameter circle centered on the plot within the QGIS (*66*) and ArcGIS (*72*) software environments. Each wildfire field plot was paired with an identically sized, nearby (between 15 to 150 m outside the burn scar) unburned forest to serve as an estimate of prefire conditions. Locations of burned sites and their paired controls were adjusted to best match several spatially-explicit datasets considered correlative of forest C storage. Overstory biomass, dominant overstory species and stand age were best matched using 12.5 m resolution forest maps provided by the Swedish University of Agricultural Sciences (*60*). Matching of slope and minimization of elevation change between plot-pairs proceeded via data that was provided by the Swedish Mapping, Cadastral and Land Registration Authority from a 50 m resolution digital elevation model (*73*). As a complement to the quantitative overstory data, aerial and satellite images provided by the Swedish National Land Survey were examined.

This gave quick visual verification that plot pairs, and the area between them, had not been disturbed by fire or logging over the past 30 years and that stand structure appeared homogeneous and uninterrupted by roads or other artificial structures. Importantly, stands were also matched for topo-edaphically derived soil moisture (TEM) estimates. TEM was provided at 10 m resolution by the Swedish Environmental Protection Agency (*59*) as integer values ranging from 0 to 240 (in order of increasing moisture potential). TEM was based on the Soil Topographic Wetness Index (*74*) in areas where soil type information was available and on the two topographic indices Depth to Water (*75*) and the Topographic Wetness Index (*76*) where soil information was unavailable. These measures gave an estimate of soil drainage, which can explain long-term soil moisture patterns conducive of ground C storage (*19*). Stands were filtered to be greater than 30 years of age, less than 15° slope, avoid post-fire salvage logging, and inundation (i.e., TEM > 150), but were otherwise unrestricted in terms of landscape position, fire severity and fire intensity. After these filters, field sites were selected to maximize their spread across MAT and MAP gradients (*77*). MAT and MAP values were derived from daily records averaged over the period 1961-2017 that were extracted from a 4 km resolution hydrological modeling dataset provided by the Swedish Meteorological and Hydrological Institute (SMHI) (*78*). As a result, the 50 field sites spread 0.43–7.77 °C of MAT and 539–772 mm of MAP over an approximately 57–67° latitudinal change. Further detail regarding site selection is found in Reference (*19*).

For the purpose of estimating prefire C storage and its emission rate due to wildfire, all 100 sites (50 burned, 50 control) were visited approximately 1 year post fire between 5 and 20 August 2019. For each plot, C storage was estimated in both the soil and vegetation. Estimates of soil and understory vegetation C emissions were derived by subtracting burned plot C estimates from those of their paired control plots. Because the overstory was found to have minimal fire-induced blackening in burned plots, C emissions from this storage pool were assumed to be negligible.

Soil horizon depths (i.e., the distance from bottom to top of each individual layer) of the mineral, duff, moss/litter, and char layers were measured at 20 points per plot from 10 equally spaced excavations along each plot diagonal (*79*). The mineral layer was measured from its highest rock obtrusion to the bottom of the duff layer. The duff layer was considered the grouping of the F (partially decomposed material) and H (humic material) layers in accordance with the Canadian system of soil classification (*80*), as is common in boreal wildfire literature. The moss/litter layer was all unburned material on top of the duff layer, including visually identifiable detritus and living moss. Woody debris mixed in the moss/litter layer was sampled in this study, though not the coarse woody debris laying on top of this layer. While larger dead wood lying on top of the forest floor can contribute to C and N stocks and their losses due to fire, this material is typically of low prevalence in Sweden (*81*) and generally considered to contribute minimally to wildfire emissions (*19, 20*). In all burned sites, a layer of conglomerated char formed a clear boundary on top of the moss/litter allowing for distinct measurement. Here, char is defined as fully blackened, brittle material with apparent high heat exposure due to fire. This separation was made based on large observed differences in C and N concentrations in surface pyrogenic layers compared to lower residual layers in similar ecosystems (*29, 30, 82*). The organic layer was defined as the duff, moss/litter, and char layers grouped together. Bulk density was calculated by dividing dry weight by sample volume for samples of the layers at at least four points per plot. Understory vegetation mass was estimated via cutting of biomass from areas representative of each plot. C and nitrogen (N) contents of samples were determined using a Costech ECS 4010 elemental analyzer. The analyzer was equipped with a 2 m packed chromatographic column for gas separation and calibrated after every 10 samples with standardized acetanilide (provided by the company Elemental Microanalysis, Okehampton, United Kingdom) to provide accuracy within 1%. More detailed information regarding field data collection and analysis is found in Reference (*19*).

S2 Motivating usage of fire weather variables

Daily fire weather at the time of burning has theoretical potential to predict fire behavior (*16*). Higher **air temperature**, reported near its typical peak at 2:00pm, warms fuels and provides potential for lower **relative humidity** and thus more rapid fuel drying. Hotter fuel and air require less additional energy to activate the combustion reaction. Lower cumulative **precipitation** over the day also leads to drier fuels and thus less energy dissipation into heating H_2O instead of releasing heat alongside combustion products. Higher **wind speed** provides removal of non-reactive combustion products and the replenishment of oxygen, as well as driving heat transfer towards fresh fuels, spreading the fire front and building fire intensity.

These fire weather parameters can be used to calculate the variables used in the Canadian Fire Weather Index system. These calculations have been empirically tuned to represent different aspects of forest drying. The **Fine Fuel Moisture Code (FFMC)** indicates the moisture content of litter on the forest floor. Once thoroughly dried, this litter is easily ignitable and can spread flaming combustion across the ground surface. Combined with wind speed, this variable informs the **Initial Spread Index (ISI)**, which estimates fire spread rate for a given fuel type. The **Duff Moisture Code (DMC)** indicates moisture content of organic layers just below surface litter as well as medium-sized woody material. The **Drought Code (DC)** indicates moisture content of even deeper, denser organic fuels and heavier woody material. Though more difficult to ignite, denser fuels can maintain and build heat while smoldering, leading to extensive, time-extended emissions. The DMC and DC inform the **Buildup Index**, which indicates the amount of forest floor fuel that has become available for combustion due to drying. Finally, these variables combine to form the **Fire Weather Index (FWI)** and indicate overall potential fire intensity. For more information on the development and calculation of these indices see Reference (*44*).

In addition to these parameters, a variable developed specifically for the region called **HBV** was used. Similar to DMC and DC, HBV predicts moisture conditions in the deeper portions of organic soil layers, though does so using modeled moisture holding capacity of those specific layers in addition to usage of weather parameters and thus is expected to be more accurate in determining flammability of these fuel portions (*62*). The variable **date-of-burn (DOB)** was used for comparability with other studies that attempt to predict boreal wildfire emissions, under the assumption that during a continuous drought later dates in the year will have had more time to dry and thereby produce more flammable fuels.

S3 Climate and drainage as predictors of soil organic layer carbon emissions

The topo-edaphically derived soil moisture potential (TEM) metric used in this study was largely derived from factors such as topography and soil type and therefore considered to be an essentially static property of each stand. The variables Mean annual temperature (MAT) and mean annual precipitation (MAP), however, averaged heat and moisture conditions over and across years, and so their predictive power over fuel loading therefore would depend on choice of averaging period. This is especially true considering ongoing climate change, which can not only influence trends in MAT and MAP but also weather variability within growing seasons. While it may be possible to produce better fitting models of fuel accumulation using more time-sensitive and mechanistic information on moisture interaction with the specific vegetation types in each burnt stand, this data is complicated to produce, especially when requiring intensive and spatially explicit monitoring of ecosystem structure. Furthermore, such fine-scale variation in the factors that influence fuel development may be more difficult to accurately predict than regional shifts in MAT and MAP under global change. Therefore, we instead sought to demonstrate that the bulk of fuel load mediated emissions variation can be explained by fuel

load equilibration with long-term climate conditions over a large region. Figure S1A shows a relatively large degree of variability in predictive power (R^2 varying greater than 0.3) of MAT, MAP and TEM on ground carbon emissions from the 19 high-intensity fire plots when only averaging MAT and MAP over the first few years before fire, likely due to seasonal fluctuations in temperature and moisture from longer term trends. However, the predictive relationship stabilizes over longer periods of averaging, namely soon after the 30 year mark (R^2 varying less than 0.025). Figure S1B shows a relatively small fluctuation in R^2 values (< 0.03), when averaging climate values from 1961 to between 1 and 10 years before the 2018 fires. While there is a degree of subjectivity in the choice of averaging period, we conclude that when averaging climate variables across a substantial portion of forest age (in our plots min: 47, max: 146, median: 93 years), their equilibration with emissions is stable enough to allow a single regression equation to provide robust intraregional and interseasonal comparison of spatial emissions variation at least within a decade. Given that there is unknown performance of our equation to separate ecosystem types, we encourage area- and time-specific field validation and/or calibration should similar empirical modeling approaches be applied to separate regions and fire seasons.

S4 Investigating the usefulness of current carbon mapping efforts in estimating wildfire emissions

10 m resolution forest soil organic carbon (SOC) maps for Sweden were tested for their ability to be used in emissions modeling. These maps were produced according to Reference (*83*) and downloaded from the Swedish University of Agricultural Science website (*84*). Additionally, 2 m resolution organic layer depth maps were tested to correlate to organic layer C in the current study's control plots (*85*).

The modeled SOC values from Reference (*83*) did not significantly correlate with the duff

layer (p = 0.138), moss/litter layer (p = 0.436), or the entire organic layer (p = 0.195) C values at the locations of our 50 sampled control plots. However, modeled SOC values formed a significant relationship to the < 4 mm sieved duff fraction of the field control plots (p = 0.034, R^2 = 0.092). Although, our average field-sampled value of 1.73 kg C m^{-2} of this fine fraction was significantly different than that modeled at 2.19 kg C m^{-2}. This modeled value was almost half the average 4.20 kg C m^{-2} in the entire organic layer in our field-sampled control plots. Modeled SOC values were not significantly different between the locations of our paired control and burned field plots (p = 0.887).

In our control field plots the sieved duff C (1.73 kg C m^{-2}) was not significantly different (p = 0.057) from that in burned plots (1.20 kg C m^{-2}). But the litter (unburned: 0.785 kg C m^{-2}, burned: 0.230 kg C m^{-2}) and coarse duff fraction were (unburned: 1.63 kg C m^{-2}, burned: 1.04 kg C m^{-2}, p = 0.024). Therefore, the sieved humic fraction used to derive the SOC maps appeared to not be representative of the larger amount of pre-fire fuel and also the least fire-labile ground fuel portion.

The organic layer thickness map from Reference (*85*) did not significantly correlate with C in duff layers (p = 0.246) or the entire organic layer (p = 0.233) across our 50 field-sampled control plots. The moisture maps that were used to calculate the organic layer thickness in Reference (*85*) did not improve any analysis in the current study when replacing TEM.

S5 Spatially explicit landscape analysis reveals interactive anthropogenic effects on fire regime characteristics

The 10 m resolution map of wildfire burned area and emissions produced by the current study, when combined with available forest geospatial data, allowed for exceptionally detailed comparison of the interaction of fire behavior with landscape structure. This section explores the potential role of several anthropogenic factors in shaping the region's fire regime.

First, FRP signal generation above the 50 sampled burned forest plots was driven positively by fire size and wind speed and negatively by overstory biomass and site drainage, as described in the main text. The suppressive effect of overstory biomass on the FRP signal may result from occlusion of heat radiation by the forest canopy or structural density of fire-resisting trees, such as *Pinus sylvestris*, which can limit air flow, maintain humidity, and sink heat while providing limited release of additional combustion energy to propagate the fire (*86*). Interestingly, a relatively high proportion of burning occurred in young forests during 2018 (fig. S2D). This trend is likely due the majority of stand replacement in Sweden being due to clear-cutting rather than fire. The harvesting process can add debris to the remaining fresh ground fuel while replacing the canopy with young, even-aged conifers. These highly connected and flammable fuel loads, spread across large open areas, may have been important for allowing the development of rapid wind speed and fire spread, generating high-intensity burning capable of moving fire into wetter and more dense, fire-resisting vegetation. Indeed, fig. S2C and E demonstrate disproportionate burning in poorly drained soils and high biomass stands, despite their lower observed propensity to propagate high-intensity fire. One possible explanation is that these land cover types experienced higher burn rates than expected due to their proximity to young, clear-cut forests. This pattern resembles the secondary ignition observed during the smoldering combustion phase in organic soils, as discussed above. However, in this case, the mechanism may involve the horizontal spread of wildfire heat across different forest structure at the landscape scale, rather than vertical spread within a single stand. These results present a hypothesis that clear-cutting practices could drive intensification of fire regimes which increases the vulnerability of adjacent areas with high fuel loading. Further investigation, with particular attention to the accuracy of geospatial datasets, is needed to quantify this potential effect.

Second, because the measured negative influence of TEM on FRP signal generation implies that well-drained areas burned more intensely, artificially drained areas are likely particu-

larly prone to high combustion rates. This would be due to longer term accumulation of large amounts of combustible material under inundation (*87*), with an abrupt transition in moisture dynamics after draining that could allow combustion to be more limited by fuel availability than moisture content (*88, 89*). An illustrative example of this phenomena is the extensive 131 km^2 Sala fire occurring in Sweden during 2014, which traveled through a diverse landscape of upland forests (intermediate emissions) interspersed with drained (highest emissions) and undrained peatlands (lowest emissions) (*20*). Analysis of 2018 fire activity at the national level revealed a proportionally high occurrence of fire in areas characterized by poor drainage (i.e., higher TEM, fig. S2c). Although, the TEM metric does not specifically delineate artificially drained areas, indicating that a notable amount of this burning may have occurred in drained peatlands. Therefore, to accurately assess both landscape-level C storage and the potential impact of fire on C dynamics, regional-scale analyses that specifically focus on the unnatural moisture dynamics of artificially drained areas are warranted. Such studies will not only improve understanding of overall C fluxes in response to fire but also inform land management practices aimed at mitigating C loss from these sensitive ecosystems (*52, 90*).

Lastly, in contrast to the complexity of forest clear cutting and peatland drainage discussed above, simple relationships between county population density and the 2018 fire season were found. County land area and populations in 2018 were extracted from the database of the government agency Statistics Sweden (*91*). Population density was calculated by dividing county population by its area (Pop. km^{-2}). The natural log of county population density related to the natural log of the average fire size (r = -0.758) and the natural log of the ratio of high-intensity burned area to low intensity burned area (p = 0.003, r = -0.726), demonstrating a decrease in fire intensity and spread with human population. The strong negative relationships between county population density to its average fire size and ratio of high to low intensity burned area signaled that anthropogenic factors (e.g., early warning, fire fighting, and artificial

fire breaks such as roadways, heterogeneous land use) may have been important not only in reducing burned area of a given fire event, but preventing its development into high-intensity burning. In total, 77% of fire events did not reach above half their area burning under high intensity. Without these specific mitigative human influences, it is likely that a greater portion of fires would have expanded to larger, difficult-to-control, high-intensity burns, contributing to enhanced overall burned area and C emissions during the 2018 fire season. More detailed investigation into these anthropogenic effects may lead to improved insight into controlling fire spread, potentially granting enhanced ability to provide more targeted prevention of the ignition of areas with high fuel loading and thereby better maintaining landscape C storage under intensifying fire regimes (*4, 5, 92*).

S6 Improved fuel load mapping is a key step forward in wildfire emissions modeling

While providing improved spatial resolution and statistical constraint in the mapping capabilities of boreal ground fire emissions, the modeling approach used in the current study reveals several areas of refinement required for better overall C accounting in boreal wildfire research. External validation of our 10 m C emissions map found the 4.5 kg C m^{-2} emitted by the mixture of dryer upland forests and peatlands estimated during previous study of the 2014 Sala fire (*20*) to be captured within 15.7% (3.79 kg C m^{-2}). While this underestimate could be partly due to the high emissions in drained peatlands not captured by the upscaled model, it could also be due to its systematic error in field plot-pair matching, where lower ends of fuel loading in Equation 1 produced negative emissions. For example, the cold and relatively dry county of Norrbotten was estimated to emit -13,233 tonnes of C (table S1). In contrast, exceptionally large areal emission rates of 9.90 kg C m^{-2} were produced in the high precipitation county of Västra Götaland (fig. S4), potentially due to a drop off from linearity of emissions response at

higher ends of moisture that were not covered by field sampling (Figures 1, 2). However, low burned area in this county contributed less than 0.001% to total emissions, meaning this uncalibrated moisture range likely did not compensate for potential underestimates in the remaining burned area.

Accurate regional C maps have the potential to reduce systematic error in wildfire plot-pair matching as well as offer better constraints on emissions by replacing the less direct estimates provided by climate and site drainage (*19*). However, despite offering cross- and external-validation at 10 m resolution, soil C maps that are currently available for Sweden focus on specific soil C fractions that were not found to correlate to the larger fuel load of the forest floor of the 50 sampled control plots in the current study (*83, 93*), as discussed in Section S4. Even if current monitoring efforts were extended to the larger extent of combustible material found in boreal forests, equations that calculate emissions rates from total fuel loading will likely require consistent updating to be representative of shifting fuel structure, fire intervals and anthropogenic influence (e.g., interruption to natural moisture dynamics) under the coming decades of expected rapid global change. Shifting fire regimes are also likely to provide additional uncertainties via their increasing influence on unstudied ecosystem types such as wetlands and even recently burned forests (*94–96*). Furthermore, the current study demonstrates currently difficult to predict C retention rates under low-intensity fire. This preservation of soil organic layers can provide a greater provisioning of mobilized nutrients and reproductive material to surrounding landscapes, buffering the more ecologically devastating impacts of higher intensity fire (*45, 47*). The residual C in these areas also have greater potential for postfire C emissions or even delays in ecosystem recovery under climatic disequilibrium, further outsizing their per area impact on landscape biogeochemical cycling (*19, 49*). In addition to introducing a novel emissions upscaling method, the current study demonstrates the power and importance of rapid field sampling capable of providing regional monitoring of C stores and their climate-controlled fluxes. Im-

plementation of this methodology in a variety of burned areas is encouraged alongside existing forest monitoring protocols as well as by local research institutions or citizen science. Distributed effort can help provide broad, up-to-date surveys of shifting regional C cycling capable of determining the connection of higher-level landscape characteristics, such as pyrodiversity, to the longer-term resilience of its C stores under global change.

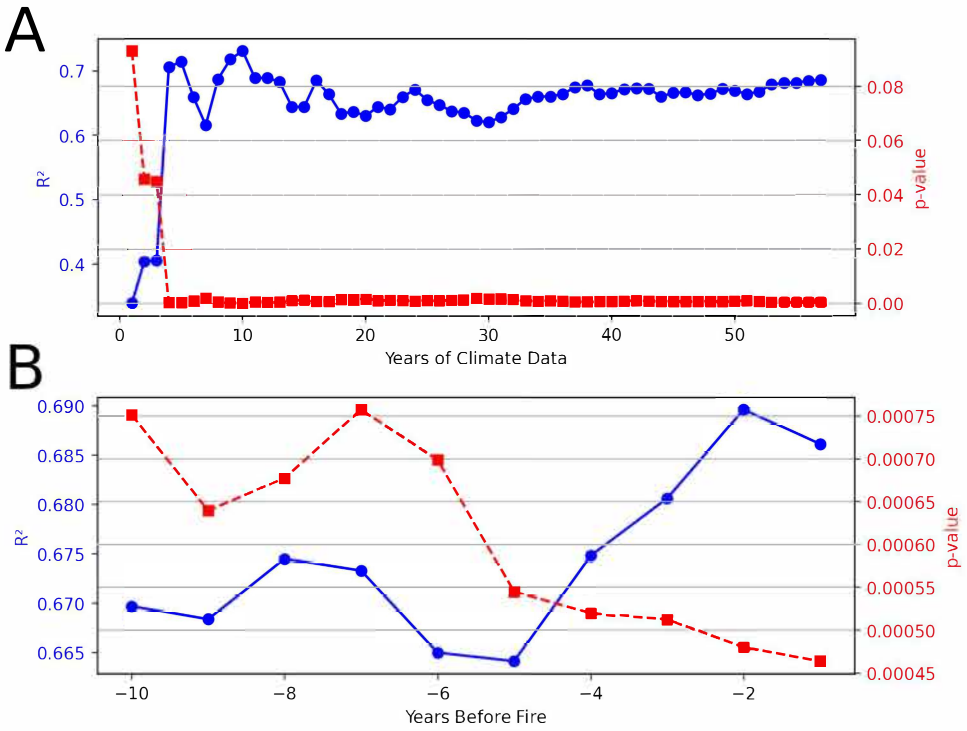

Figure S1: R^2 **and** p **values for explaining ground carbon emissions in the 19 high-intensity fire plots using their respective mean annual temperature (MAT), and mean annual precipitation (MAP) and topo-edaphically derived moisture potential (TEM) values in multiple regression. (A)** The x-axis represents the amount of years preceding the fire that MAT and MAP were averaged. An initial variability in model fit became relatively stable soon after the 30 year mark. **(B)** The climate period was averaged from 1961 to the number of years before the 2018 fires indicated by the x-axis. R^2 values varied less than by 0.025 over the 10 years shown.

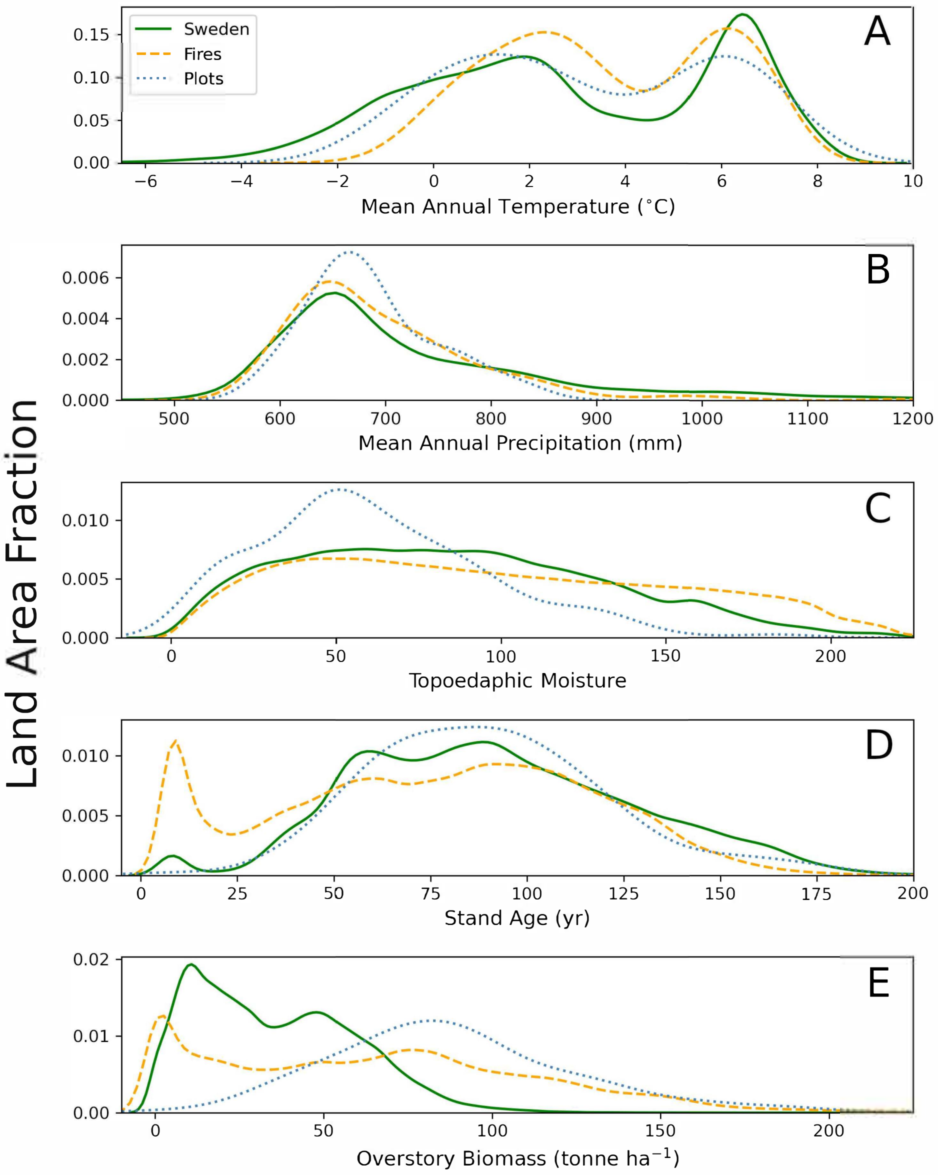

Figure S2: **Kernel density estimation plots for pixel values of available forest raster data.** Mean annual temperature **(A)**, mean annual precipitation **(B)**, topoedaphic moisture **(C)**, stand age **(D)**, and overstory biomass **(E)** are charted. Values are separated by their occurrence within the total land area of Sweden, within the 324 fire perimeters formed in 2018, and the 50 field-sampled 20 × 20 m burned plots.

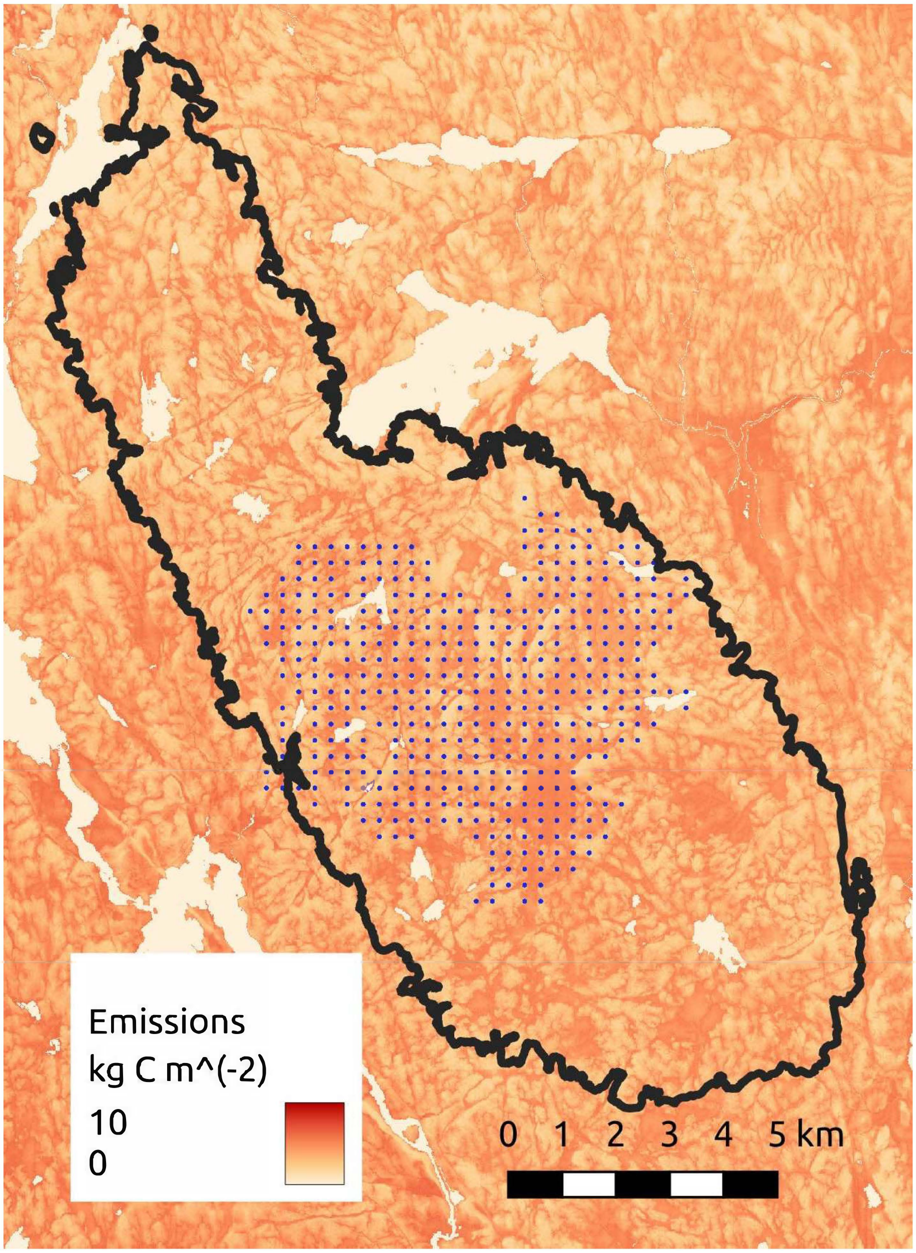

Figure S3: **Borders of the 131 km^2 Sala fire complex occurring in central Sweden during 2014, shown in black.** 10 m radius field-sampled plots are inlaid in blue. The upscaled carbon (C) emissions map from our study covers the figure, estimating the fire to release 0.487 Tg C. This is equivalent to 96% of C released from the 229 km^2 area burned in Sweden during 2018. For comparison, the largest fire complex from 2018 burned near the town of Ljusdal and was estimated to release only 21.3% of 2018 emissions over 90 km^2 (1.20 kg C m^{-2}). As shown, the mapping approach allows for the estimation of potential emission rates in unburned areas, which can be used to plan landscape structure that directs fire away from emissions hotspots.

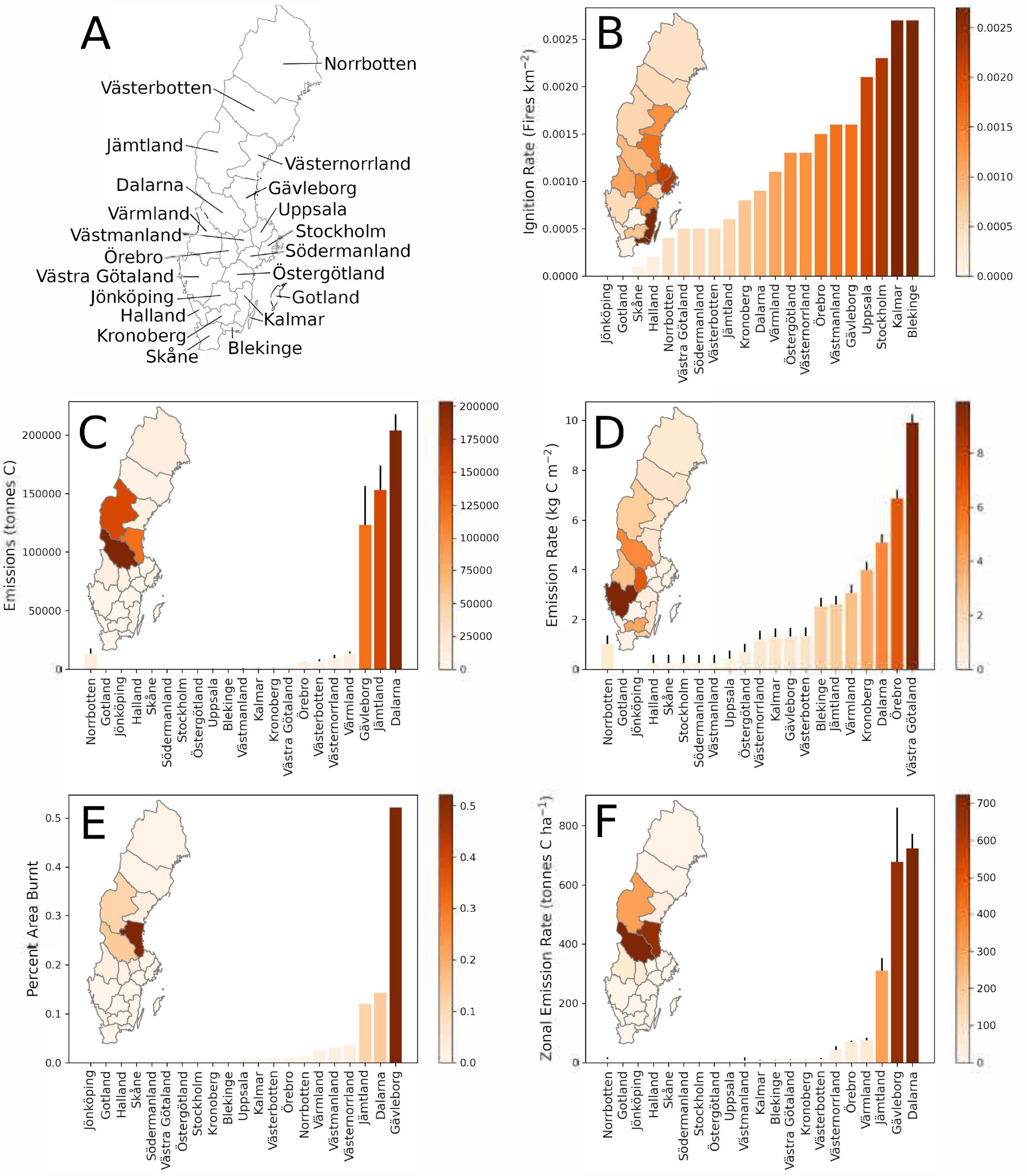

Figure S4: **Bar charts for the results of the upscaling approach found in table S1.** Each bar is colored to its corresponding county on a map of Sweden (**A**) for the areal density of fire events (**B**), total emissions (**C**), emission rate from burned areas (**D**), percentage of county area burned (**E**), and emission rate from total county area (**F**). Emissions, Emission rate, and zonal emission rate for the cold and dry Norrbotten country are negative values due to systemic model error.

A

	p	R^2	R^2_{adj}	ΔAICc	ΔBIC	BurnArea	WindSpeed	OverstoryBM	TEM	StandAge	MAP
1	0.0	0.561753	0.561753	6.404161	1.473648	3.464330	NaN	NaN	NaN	NaN	NaN
2	0.0	0.618585	0.618585	4.802139	1.611663	4.425979	1.027456	NaN	NaN	NaN	NaN
3	0.0	0.65813	0.65813	4.442512	2.897639	5.237749	1.482628	-0.900595	NaN	NaN	NaN
4	0.0	0.760676	0.760676	0.0	0.0	8.622630	2.958647	-2.135476	-2.035607	NaN	NaN
5	0.0	0.782289	0.782289	1.039452	2.476728	9.979580	3.681866	-2.424318	-2.195998	-0.867592	NaN
6	0.0	0.835569	0.835569	0.091232	2.850678	13.909829	4.869028	-2.746760	-2.859764	-1.949187	-1.88312

B

	p	R^2	R^2_{adj}	ΔAICc	ΔBIC	SOLC	Weather5	Weather3	Weather1	Weather2	Weather4
1	0.000002	0.748434	0.733637	0.0	0.0	0.825296	NaN	NaN	NaN	NaN	NaN
2	0.000007	0.774682	0.746517	0.42109	0.850823	0.807233	0.591861	NaN	NaN	NaN	NaN
3	0.00003	0.783875	0.74065	2.479604	3.003776	0.794270	0.597585	-0.200099	NaN	NaN	NaN
4	0.000108	0.792666	0.733428	4.947778	5.159247	0.737952	0.622451	-0.216990	0.220007	NaN	NaN
5	0.000419	0.793999	0.714768	8.583483	7.981148	0.726704	0.627418	-0.220363	0.232188	-0.063665	NaN
6	0.001432	0.794512	0.691768	12.920685	10.878174	0.715247	0.632476	-0.223799	0.244595	-0.068073	-0.103161

C

	p	R^2	R^2_{adj}	ΔAICc	ΔBIC	SOLC	Weather5	Weather2	Weather1	Weather3	Weather4
1	0.003739	0.255316	0.229637	3.600885	1.503507	0.544395	NaN	NaN	NaN	NaN	NaN
2	0.001735	0.364961	0.319601	0.954032	0.0	0.625572	0.840805	NaN	NaN	NaN	NaN
3	0.001437	0.43119	0.367989	0.0	0.019638	0.656432	0.856540	-0.313240	NaN	NaN	NaN
4	0.002115	0.464103	0.381657	0.801858	1.605911	0.581194	0.818178	-0.304350	-0.326434	NaN	NaN
5	0.003269	0.489819	0.387782	2.138949	3.51545	0.560317	0.807533	-0.301883	-0.337505	-0.248258	NaN
6	0.007096	0.495492	0.369365	4.892272	6.60276	0.543488	0.798952	-0.299894	-0.346429	-0.250639	-0.104657

Figure S5: **Tabulated regression model selection results.** (**A**) Logistic regression model selection for explaining FRP signal presence as a boolean using variables in Table 1. Multiple regression model selection for explaining SOLC emissions in high-intensity (**B**) and low-intensity (**C**) fire using partial least squares regression derived latent variables from the categories *Fire Weather Parameters* and *Canadian Fire Weather Index* in Table 1. Values in the variable columns are the standardized regression coefficients β.

Zone	NUTS	Pop. Density (Pop. km^{-2})	Fires	Ignition Rate (Fires km^{-2})	Fire Size (ha)	Burn Area (km^2)	Burn Area %	Emissions (tonnes C)	Emission Rate (kg C m^{-2})	Zonal Emission Rate (tonnes C ha^{-1})
Sweden	SE	25.5	324	0.0008	70.6	228.79	0.0558	507826 $\pm$ 79993	2.22 $\pm$ 0.35	123.776 $\pm$ 19.497
**Östra Sverige**	SE1	92.4	70	0.0016	4.5	3.16	0.0070	6740 $\pm$ 1051	2.13 $\pm$ 0.33	14.937 $\pm$ 2.329
Stockholm	SE11	370.5	15	0.0023	0.8	0.13	0.0020	33 $\pm$ 41	0.26 $\pm$ 0.32	0.503 $\pm$ 0.635
Stockholm	SE110	370.5	15	0.0023	0.8	0.13	0.0020	33 $\pm$ 41	0.26 $\pm$ 0.32	0.503 $\pm$ 0.635
Östra Mellansverige	SE12	45.4	55	0.0014	5.5	3.04	0.0079	6707 $\pm$ 1010	2.21 $\pm$ 0.33	17.375 $\pm$ 2.615
Uppsala	SE121	48.1	17	0.0021	2.4	0.40	0.0049	168 $\pm$ 134	0.42 $\pm$ 0.33	2.053 $\pm$ 1.63
Södermanland	SE122	49.5	3	0.0005	1.1	0.03	0.0005	8 $\pm$ 10	0.26 $\pm$ 0.32	0.134 $\pm$ 0.168
Östergötland	SE123	44.3	14	0.0013	1.1	0.16	0.0015	110 $\pm$ 52	0.69 $\pm$ 0.33	1.037 $\pm$ 0.493
Örebro	SE124	35.9	13	0.0015	6.7	0.88	0.0103	6017 $\pm$ 304	6.86 $\pm$ 0.35	70.408 $\pm$ 3.558
Västmanland	SE125	54.2	8	0.0016	19.6	1.57	0.0305	404 $\pm$ 509	0.26 $\pm$ 0.32	7.846 $\pm$ 9.893
**Södra Sverige**	SE2	59.0	58	0.0008	2.3	1.32	0.0017	4749 $\pm$ 446	3.6 $\pm$ 0.34	6.19 $\pm$ 0.581
Småland med öarna	SE21	26.4	37	0.0011	2.5	0.94	0.0028	1894 $\pm$ 318	2.01 $\pm$ 0.34	5.681 $\pm$ 0.953
Jönköping	SE211	35.0	0	0.0000	0.0	0.00	0.0000	0	0	0
Kronoberg	SE212	24.0	7	0.0008	3.6	0.25	0.0030	1006 $\pm$ 87	3.97 $\pm$ 0.34	11.888 $\pm$ 1.024
Kalmar	SE213	22.0	30	0.0027	2.3	0.69	0.0061	887 $\pm$ 231	1.29 $\pm$ 0.34	7.909 $\pm$ 2.059
Gotland	SE214	19.4	0	0.0000	0.0	0.00	0.0000	0	0	0
Sydsverige	SE22	111.7	9	0.0006	1.2	0.11	0.0008	261 $\pm$ 37	2.35 $\pm$ 0.34	1.864 $\pm$ 0.266
Blekinge	SE221	53.9	8	0.0027	1.3	0.10	0.0035	258 $\pm$ 34	2.52 $\pm$ 0.34	8.773 $\pm$ 1.167
Skåne	SE224	127.1	1	0.0001	0.8	0.01	0.0001	2 $\pm$ 3	0.26 $\pm$ 0.32	0.02 $\pm$ 0.025
Västsverige	SE23	70.9	12	0.0004	2.2	0.27	0.0009	2595 $\pm$ 91	9.74 $\pm$ 0.34	8.822 $\pm$ 0.311
Halland	SE231	62.3	1	0.0002	0.4	0.00	0.0001	1 $\pm$ 1	0.26 $\pm$ 0.32	0.02 $\pm$ 0.026
Västra Götaland	SE232	72.9	11	0.0005	2.4	0.26	0.0011	2593 $\pm$ 90	9.9 $\pm$ 0.34	10.829 $\pm$ 0.376
**Norra Sverige**	SE3	6.1	196	0.0007	114.4	224.31	0.0778	496338 $\pm$ 78496	2.21 $\pm$ 0.35	172.08 $\pm$ 27.214
Norra Mellansverige	SE31	13.4	75	0.0012	185.8	139.37	0.2178	340374 $\pm$ 48755	2.44 $\pm$ 0.35	532.011 $\pm$ 76.204
Värmland	SE311	16.1	20	0.0011	21.9	4.38	0.0249	13388 $\pm$ 1474	3.06 $\pm$ 0.34	76.109 $\pm$ 8.378
Dalarna	SE312	10.2	26	0.0009	154.2	40.09	0.1422	203734 $\pm$ 14008	5.08 $\pm$ 0.35	722.747 $\pm$ 49.694
Gävleborg	SE313	15.8	29	0.0016	327.2	94.90	0.5215	123252 $\pm$ 33273	1.3 $\pm$ 0.35	677.249 $\pm$ 182.827
Mellersta Norrland	SE32	5.3	56	0.0008	119.3	66.83	0.0941	162523 $\pm$ 23418	2.43 $\pm$ 0.35	228.825 $\pm$ 32.971
Västernorrland	SE321	11.3	28	0.0013	28.0	7.84	0.0361	9404 $\pm$ 2712	1.2 $\pm$ 0.35	43.367 $\pm$ 12.508
Jämtland	SE322	2.7	28	0.0006	210.7	59.00	0.1196	153119 $\pm$ 20706	2.6 $\pm$ 0.35	310.327 $\pm$ 41.964
Övre Norrland	SE33	3.4	65	0.0004	27.9	18.11	0.0118	-6559 $\pm$ 6323	-0.36 $\pm$ 0.35	-4.275 $\pm$ 4.121
Västerbotten	SE331	5.0	26	0.0005	19.2	4.99	0.0090	6674 $\pm$ 1742	1.34 $\pm$ 0.35	12.093 $\pm$ 3.156
Norrbotten	SE332	2.5	39	0.0004	33.6	13.12	0.0134	-13233 $\pm$ 4581	-1.01 $\pm$ 0.35	-13.47 $\pm$ 4.663

Table S1: **Upscaled results for the 2018 fire season in Sweden divided by Nomenclature of Territorial Units for Statistics (NUTS) zones.**

REFERENCES

1. C. J. A. Bradshaw, I. G. Warkentin, Global estimates of boreal forest carbon stocks and flux. *Glob. Planet. Change* **128**, 24–30 (2015).

2. P. Friedlingstein, M. O'Sullivan, M. W. Jones, R. M. Andrew, D. C. E. Bakker, J. Hauck, P. Landschützer, C. L. Quéré, I. T. Luijkx, G. P. Peters, W. Peters, J. Pongratz, C. Schwingshackl, S. Sitch, J. G. Canadell, P. Ciais, R. B. Jackson, S. R. Alin, P. Anthoni, L. Barbero, N. R. Bates, M. Becker, N. Bellouin, B. Decharme, L. Bopp, I. B. M. Brasika, P. Cadule, M. A. Chamberlain, N. Chandra, T.-T.-T. Chau, F. Chevallier, L. P. Chini, M. Cronin, X. Dou, K. Enyo, W. Evans, S. Falk, R. A. Feely, L. Feng, D. J. Ford, T. Gasser, J. Ghattas, T. Gkritzalis, G. Grassi, L. Gregor, N. Gruber, Ö. Gürses, I. Harris, M. Hefner, J. Heinke, R. A. Houghton, G. C. Hurtt, Y. Iida, T. Ilyina, A. R. Jacobson, A. Jain, T. Jarníková, A. Jersild, F. Jiang, Z. Jin, F. Joos, E. Kato, R. F. Keeling, D. Kennedy, K. K. Goldewijk, J. Knauer, J. I. Korsbakken, A. Körtzinger, X. Lan, N. Lefèvre, H. Li, J. Liu, Z. Liu, L. Ma, G. Marland, N. Mayot, P. C. Mc Guire, G. A. Mc Kinley, G. Meyer, E. J. Morgan, D. R. Munro, S.-I. Nakaoka, Y. Niwa, K. M. O'Brien, A. Olsen, A. M. Omar, T. Ono, M. Paulsen, D. Pierrot, K. Pocock, B. Poulter, C. M. Powis, G. Rehder, L. Resplandy, E. Robertson, C. Rödenbeck, T. M. Rosan, J. Schwinger, R. Séférian, T. L. Smallman, S. M. Smith, R. Sospedra-Alfonso, Q. Sun, A. J. Sutton, C. Sweeney, S. Takao, P. P. Tans, H. Tian, B. Tilbrook, H. Tsujino, F. Tubiello, G. R. van der Werf, E. van Ooijen, R. Wanninkhof, M. Watanabe, C. Wimart-Rousseau, D. Yang, X. Yang, W. Yuan, X. Yue, S. Zaehle, J. Zeng, B. Zheng, Global carbon budget 2023. *Earth Syst. Sci. Data* **15**, 5301–5369 (2023).

3. M. W. Jones, M. W. Jones, S. Veraverbeke, N. Andela, S. H. Doerr, C. Kolden, G. Mataveli, M. Lucrecia Pettinari, C. Le Quéré, T. M. Rosan, G. R. van der Werf, D. van Wees, J. T. Abatzoglou, Global rise in forest fire emissions linked to climate change in the extratropics. *Science* **386**, eadl5889 (2024).

4. R. Kelly, M. L. Chipman, P. E. Higuera, I. Stefanova, L. B. Brubaker, F. S. Hu, Recent burning of boreal forests exceeds fire regime limits of the past 10,000 years. *Proc. Natl. Acad. Sci. U.S.A.* **110**, 13055–13060 (2013).

5. W. J. De Groot, P. M. Bothwell, D. Carlsson, K. A. Logan, Simulating the effects of future fire regimes on western canadian boreal forests. *J. Veg. Sci.* **14**, 355–364 (2003).

6. B. Zheng, P. Ciais, F. Chevallier, H. Yang, J. G. Canadell, Y. Chen, I. R. van der Velde, I. Aben, E. Chuvieco, S. J. Davis, M. Deeter, C. Hong, Y. Kong, H. Li, H. Li, X. Lin, K. He, Q. Zhang, Record-high CO_2 emissions from boreal fires in 2021. *Science* **379**, 912–917 (2023).

7. S. Veraverbeke, C. J. F. Delcourt, E. Kukavskaya, M. Mack, X. Walker, T. Hessilt, B. Rogers, R. C. Scholten, Direct and longer-term carbon emissions from arctic-boreal fires: A short review of recent advances. *Curr. Opin. Environ. Sci. Health* **23**, 100277 (2021).

8. K. Barrett, E. S. Kasischke, Controls on variations in MODIS fire radiative power in alaskan boreal forests: Implications for fire severity conditions. *Remote Sens. Environ.* **130**, 171–181 (2013).

9. E. Vermote, E. Ellicott, O. Dubovik, T. Lapyonok, M. Chin, L. Giglio, G. J. Roberts, An approach to estimate global biomass burning emissions of organic and black carbon from MODIS fire radiative power. *J. Geophys. Res.* **114**, D18205 (2009).

10. S. Potter, S. Cooperdock, S. Veraverbeke, X. Walker, M. C. Mack, S. J. Goetz, J. Baltzer, L. Bourgeau-Chavez, A. Burrell, C. Dieleman, N. French, S. Hantson, E. E. Hoy, L. Jenkins, J. F. Johnstone, E. S. Kane, S. M. Natali, J. T. Randerson, M. R. Turetsky, E. Whitman, E. Wiggins, B. M. Rogers, Burned area and carbon emissions across northwestern boreal North America from 2001–2019. *Biogeosciences* **20**, 2785–2804 (2023).

11. S. Veraverbeke, B. M. Rogers, J. T. Randerson, Daily burned area and carbon emissions from boreal fires in Alaska. *Biogeosciences* **12**, 3579–3601 (2015).

12. B. M. Rogers, S. Veraverbeke, G. Azzari, C. I. Czimczik, S. R. Holden, G. O. Mouteva, F. Sedano, K. K. Treseder, J. T. Randerson, Quantifying fire-wide carbon emissions in interior Alaska using field measurements and Landsat imagery. *J. Geophys. Res. Biogeosci.* **119**, 1608–1629 (2014).

13. D. van Wees, G. R. van der Werf, J. T. Randerson, B. M. Rogers, Y. Chen, S. Veraverbeke, L. Giglio, D. C. Morton, Global biomass burning fuel consumption and emissions at 500 m spatial resolution based on the global fire emissions database (GFED). *Geosci. Model Dev.* **15**, 8411–8437 (2022).

14. B. Byrne, J. Liu, K. W. Bowman, M. Pascolini-Campbell, A. Chatterjee, S. Pandey, K. Miyazaki, G. van der Werf, D. Wunch, P. O. Wennberg, C. M. Roehl, S. Sinha, Carbon emissions from the 2023 Canadian wildfires. *Nature* **633**, 835–839 (2024).

15. C. A. Phillips, B. M. Rogers, M. Elder, S. Cooperdock, M. Moubarak, J. T. Randerson, P. C. Frumhoff, Escalating carbon emissions from North American boreal forest wildfires and the climate mitigation potential of fire management. *Sci. Adv.* **8**, eabl7161 (2022).

16. B. J. Stocks, B. D. Lawson, M. E. Alexander, C. E. Van Wagner, R. S. McAlpine, T. J. Lynham, D. E. Dubé, The canadian forest fire danger rating system: An overview. *For. Chron.* **65**, 450–457 (1989).

17. M.-A. Parisien, S. A. Parks, C. Miller, M. A. Krawchuk, M. Heathcott, M. A. Moritz, Contributions of ignitions, fuels, and weather to the spatial patterns of burn probability of a boreal landscape. *Ecosystems* **14**, 1141–1155 (2011).

18. X. Walker, B. M. Rogers, S. Veraverbeke, J. F. Johnstone, J. L. Baltzer, K. Barrett, L. Bourgeau-Chavez, N. J. Day, W. J. de Groot, C. M. Dieleman, S. Goetz, E. Hoy, L. K. Jenkins, E. S. Kane, M.-A. Parisien, S. Potter, E. A. G. Schuur, M. Turetsky, E. Whitman, M. C. Mack, Fuel availability not fire weather controls boreal wildfire severity and carbon emissions. *Nat. Clim. Chang.* **10**, 1130–1136 (2020).

19. J. A. Eckdahl, J. A. Kristensen, D. B. Metcalfe, Climatic variation drives loss and restructuring of carbon and nitrogen in boreal forest wildfire. *Biogeosciences* **19**, 2487–2506 (2022).

20. G. Granath, C. D. Evans, J. Strengbom, J. Fölster, A. Grelle, J. Strömqvist, S. J. Köhler, The impact of wildfire on biogeochemical fluxes and water quality in boreal catchments. *Biogeosciences* **18**, 3243–3261 (2021).

21. L. Giglio, W. Schroeder, C. O. Justice, The collection 6 MODIS active fire detection algorithm and fire products. *Remote Sens. Environ.* **178**, 31–41 (2016).

22. K. Miyanishi, E. A. Johnson, Process and patterns of duff consumption in the mixedwood boreal forest. *Can. J. For. Res.* **32**, 1285–1295 (2002).

23. G. Rein, *Smouldering Fires and Natural Fuels* (John Wiley& Sons Ltd., 2013), chap. 2, pp. 15–33.

24. R. C. Scholten, R. Jandt, E. A. Miller, B. M. Rogers, S. Veraverbeke, Overwintering fires in boreal forests. *Nature* **593**, 399–404 (2021).

25. B. Bond-Lamberty, S. D. Peckham, D. E. Ahl, S. T. Gower, Fire as the dominant driver of central canadian boreal forest carbon balance. *Nature* **450**, 89–92 (2007).

26. L. Boschetti, D. P. Roy, Strategies for the fusion of satellite fire radiative power with burned area data for fire radiative energy derivation. *J. Geophys. Res.* **114**, D20302 (2009).

27. P. Vanhala, K. Karhu, M. Tuomi, K. Björklöf, H. Fritze, J. Liski, Temperature sensitivity of soil organic matter decomposition in southern and northern areas of the boreal forest zone. *Soil Biol. Biochem.* **40**, 1758–1764 (2008).

28. L. Kohl, M. Philben, K. A. Edwards, F. A. Podrebarac, J. Warren, S. E. Ziegler, The origin of soil organic matter controls its composition and bioreactivity across a mesic boreal forest latitudinal gradient. *Glob. Chang. Biol.* **24**, e458–e473 (2018).

29. C. Santín, S. H. Doerr, A. Merino, R. Bryant, N. J. Loader, Forest floor chemical transformations in a boreal forest fire and their correlations with temperature and heating duration. *Geoderma* **264**, 71–80 (2016).

30. A. A. Dymov, V. V. Startsev, E. Y. Milanovsky, I. A. Valdes-Korovkin, Y. R. Farkhodov, A. V. Yudina, O. Donnerhack, G. Guggenberger, Soils and soil organic matter transformations during the two years after a low-intensity surface fire (Subpolar Ural, Russia). *Geoderma* **404**, 115278 (2021).

31. M. W. Schmidt, A. G. Noack, Black carbon in soils and sediments: Analysis, distribution, implications, and current challenges. *Global Biogeochem. Cycles* **14**, 777–793 (2000).

32. J. Cornelissen, R. Aerts, B. Cerabolini, M. Werger, M. Van Der Heijden, Carbon cycling traits of plant species are linked with mycorrhizal strategy. *Oecologia* **129**, 611–619 (2001).

33. C. S. Delavaux, J. A. La Manna, J. A. Myers, R. P. Phillips, S. Aguilar, D. Allen, A. Alonso, K. J. Anderson-Teixeira, M. E. Baker, J. L. Baltzer, P. Bissiengou, M. Bonfim, N. A. Bourg, W. Y. Brockelman, D. F. R. P. Burslem, L.-W. Chang, Y. Chen, J.-M. Chiang, C. Chu, K. Clay, S. Cordell, M. Cortese, J. den Ouden, C. Dick, S. Ediriweera, E. C. Ellis, A. Feistner, A. L. Freestone, T. Giambelluca, C. P. Giardina, G. S. Gilbert, F. He, J. Holík, R. W. Howe, W. H. Huasca, S. P. Hubbell, F. Inman, P. A. Jansen, D. J. Johnson, K. Kral, A. J. Larson, C. M. Litton, J. A. Lutz, Y. Malhi, K. M. Guire, S. M. Mc Mahon, W. J. Mc Shea, H. Memiaghe, A. Nathalang, N. Norden, V. Novotny, M. J. O'Brien, D. A. Orwig, R. Ostertag, G. G. ('. J.') Parker, R. Pérez, G. Reynolds, S. E. Russo, L. Sack, P. Šamonil, I.-F. Sun, M. E. Swanson, J. Thompson, M. Uriarte, J. Vandermeer, X. Wang, I. Ware, G. D. Weiblen, A. Wolf, S.-H. Wu, J. K. Zimmerman, T. Lauber, D. S. Maynard, T. W. Crowther, C. Averill, Mycorrhizal feedbacks influence global forest structure and diversity. *Commun. Biol.* **6**, 1066 (2023).

34. B. M. Rogers, A. J. Soja, M. L. Goulden, J. T. Randerson, Influence of tree species on continental differences in boreal fires and climate feedbacks. *Nat. Geosci.* **8**, 228–234 (2015).

35. X. J. Walker, B. M. Rogers, J. L. Baltzer, S. G. Cumming, N. J. Day, S. J. Goetz, J. F. Johnstone, E. A. G. Schuur, M. R. Turetsky, M. C. Mack, Cross-scale controls on carbon emissions from boreal forest megafires. *Global Change Biol.* **24**, 4251–4265 (2018).

36. Y. Shi, T. Matsunaga, M. Saito, Y. Yamaguchi, X. Chen, Comparison of global inventories of CO_2 emissions from biomass burning during 2002–2011 derived from multiple satellite products. *Environ. Pollut.* **206**, 479–487 (2015).

37. R. C. Scholten, D. Coumou, F. Luo, S. Veraverbeke, Early snowmelt and polar jet dynamics co-influence recent extreme siberian fire seasons. *Science* **378**, 1005–1009 (2022).

38. A. Sirin, M. Medvedeva, V. Korotkov, V. Itkin, T. Minayeva, D. Ilyasov, G. Suvorov, H. Joosten, Addressing peatland rewetting in russian federation climate reporting. *Land* **10**, 1200 (2021).

39. K. Hayes, C. M. Hoffman, R. Linn, J. Ziegler, B. Buma, Fuel constraints, not fire weather conditions, limit fire behavior in reburned boreal forests. *Agric. For. Meteorol.* **358**, 110216 (2024).

40. E. Whitman, Q. E. Barber, P. Jain, S. A. Parks, L. Guindon, D. K. Thompson, M. A. Parisien, A modest increase in fire weather overcomes resistance to fire spread in recently burned boreal forests. *Glob. Change Biol.* **30**, e17363 (2024).

41. S. S. Jones, M. Matsala, E. V. Delin, N. Subramanian, U. Nilsson, E. Holmström, I. Drobyshev, Forest structure, roads and soil moisture provide realistic predictions of fire spread in modern swedish landscape. *Ecol. Model.* **499**, 110942 (2025).

42. F. Vermina Plathner, J. Sjöström, A. Granström, Garden structure is critical for building survival in northern forest fires–An analysis using large swedish wildfires. *Saf. Sci.* **157**, 105928 (2023).

43. S. P. Bowring, W. Li, F. Mouillot, T. M. Rosan, P. Ciais, Road fragment edges enhance wildfire incidence and intensity, while suppressing global burned area. *Nat. Commun.* **15**, 9176 (2024).

44. C. Van Wagner, "Development and structure of the canadian forest fireweather index system" (Canadian Forest Service Technical Report, 1987).

45. T. M. Porter, E. Smenderovac, D. Morris, L. Venier, All boreal forest successional stages needed to maintain the full suite of soil biodiversity, community composition, and function following wildfire. *Sci. Rep.* **13**, 7978 (2023).

46. Z. L. Steel, J. E. D. Miller, L. C. Ponisio, M. W. Tingley, K. Wilkin, R. Blakey, K. M. Hoffman, G. Jones, A roadmap for pyrodiversity science. *J. Biogeogr.* **51**, 280–293 (2024).

47. J. A. Eckdahl, J. A. Kristensen, D. B. Metcalfe, Climate and forest properties explain wildfire impact on microbial community and nutrient mobilization in boreal soil. *Frontiers For. Glob. Change* **6**, 1136354 (2023).

48. J. A. Eckdahl, J. A. Kristensen, D. B. Metcalfe, Restricted plant diversity limits carbon recapture after wildfire in warming boreal forests. *Commun. Earth Environ.* **5**, 186 (2024).

49. J. Kelly, N. Kljun, Z. Cai, S. H. Doerr, C. D'Onofrio, T. Holst, I. Lehner, A. Lindroth, S. Thapa, P. Vestin, C. Santín, Wildfire impacts on the carbon budget of a managed nordic boreal forest. *Agric. For. Meteorol.* **351**, 110016 (2024).

50. M. Ueyama, H. Iwata, H. Nagano, N. Tahara, C. Iwama, Y. Harazono, Carbon dioxide balance in early-successional forests after forest f ires in interior alaska. *Agric. For. Meteorol.* **275**, 196–207 (2019).

51. D. K. Thompson, B. N. Simpson, E. Whitman, Q. E. Barber, M.-A. Parisien, Peatland hydrological dynamics as a driver of landscape connectivity and fire activity in the boreal plain of canada. *Forests* **10**, 534 (2019).

52. A. Günther, A. Barthelmes, V. Huth, H. Joosten, G. Jurasinski, F. Koebsch, J. Couwenberg, Prompt rewetting of drained peatlands reduces climate warming despite methane emissions. *Nat. Commun.* **11**, 1644 (2020).

53. M. R. Turetsky, B. Benscoter, S. Page, G. Rein, G. R. van der Werf, A. Watts, Global vulnerability of peatlands to fire and carbon loss. *Nat. Geosci.* **8**, 11–14 (2015).

54. J. Schimmel, A. Granström, Fuel succession and fire behavior in the swedish boreal forest. *Can. J. For. Res.* **27**, 1207–1216 (1997).

55. T. A. J. Janssen, S. Veraverbeke, What are the limits to the growth of boreal fires? *Global Change Biol.* **31**, e70130 (2025).

56. B. Butler, R. D. Ottmar, T. S. Rupp, R. Jandt, E. Miller, K. Howard, R. Schmoll, S. Theisen, R. E. Vihnanek, D. Jimenez, Quantifying the effect of fuel reduction treat ments on fire behavior in boreal forests. *Can. J. For. Res.* **43**, 97–102 (2013).

57. J. A. Eckdahl, P. C. Rodriguez, J. A. Kristensen, D. B. Metcalfe, K. Ljung, Mineral soils are an important intermediate storage pool of black carbon in fennoscandian boreal forests. *Global Biogeochem. Cycles* **36**, e2022GB007489 (2022).

58. J. Eckdahl, "Boreal forest wildfire in a changing climate," thesis, Department of Physical Geography and Ecosystem Science (2023).

59. Naturvårdsverket, Markfuktighetsindex producerat som del av nationella marktäckedata, nmd 2018; https://catalogue.arctic-sdi.org/geonetwork/srv/api/records/cae71f45-b463-447f-804f-2847869b19b0. [Swedish Environmental Protection Agency, Soil moisture index produced as a part of the National Land Cover Data].

60. Swedish University of Agricultural Sciences Department of Forest Resource Management. Slu forest map (2015); https://www.slu.se/en/environment/statistics-and-environmental-data/environmental-data-catalogue/slu-forest-map/.

61. R. A. I. Wilcke, E. Kjellström, C. Lin, D. Matei, A. Moberg, E. Tyrlis, The extremely warm summer of 2018 in sweden–Set in a historical context. *Earth Syst. Dyn.* **11**, 1107–1121 (2020).

62. J. Seibert, S. Bergström, A retrospective on hydrological catchment modelling based on half a century with the hbv model. *Hydrol. Earth Syst. Sci.* **26**, 1371–1388 (2022).

63. F. Pedregosa, G. Varoquaux, A. Gramfort, V. Michel, B. Thirion, O. Grisel, M. Blondel, P. Prettenhofer, R. Weiss, V. Dubourg, J. Vanderplas, A. Passos, D. Cournapeau, M. Brucher, M. Perrot, É. Duchesnay, Scikit-learn: Machine learning in Python. *J. Mach. Learn. Res.* **12**, 2825 (2011).

64. L. Giglio, W. Schroeder, J. V. Hall, C. O. Justice, "Modis collection 6 and collection 6.1 active fire product user's guide" (National Aeronautical and Space Administration, 2021), p. 64.

65. NASA Goddard Space Flight Center, Modis fire radiative power (frp) data (2019), https://modis.gsfc.nasa.gov/data/dataprod/mod14.php [accessed 15 February 2024].

66. QGIS Development Team, “QGIS Geographic Information System” (QGIS Association, 2019).

67. P. Virtanen, R. Gommers, T. E. Oliphant, M. Haberland, T. Reddy, D. Cournapeau, E. Burovski, P. Peterson, W. Weckesser, J. Bright, S. J. van der Walt, M. Brett, J. Wilson, K. J. Millman, N. Mayorov, A. R. J. Nelson, E. Jones, R. Kern, E. Larson, C. J. Carey, İ. Polat, Y. Feng, E. W. Moore, J. V. Plas, D. Laxalde, J. Perktold, R. Cimrman, I. Henriksen, E. A. Quintero, C. R. Harris, A. M. Archibald, A. H. Ribeiro, F. Pedregosa, P. van Mulbregt, SciPy 1.0 Contributor, SciPy 1.0: Fundamental algorithms for scientific computing in python. *Nat. Methods* **17**, 261–272 (2020).

68. S. Seabold, J. Perktold, “statsmodels: Econometric and statistical modeling with python,” in *9th Python in Science Conference* (SciPy, 2010); https://doi.org/10.25080/Majora-92bf1922-011.

69. K. P. Burnham, D. R. Anderson, Multimodel inference: Understanding aic and bic in model selection. *Sociol. Methods Res.* **33**, 261–304 (2004).

70. L. A. Boby, E. A. G. Schuur, M. C. Mack, D. Verbyla, J. F. Johnstone, Quantifying fire severity, carbon, and nitrogen emissions in Alaska’s boreal forest. *Ecol. Appl.* **20**, 1633–1647 (2010).

71. A. L. Bataineh, B. P. Oswald, M. Bataineh, D. Unger, I.-K. Hung, D. Scognamillo, Spatial autocorrelation and pseudoreplication in fire ecology. *Fire Ecol.* **2**, 107–118 (2006).

72. Esri Inc., “ArcGIS Pro” (Esri Inc., 2019).

73. Lantmäteriet, Markhöjdmodell nedladdning, grid 50+ (2021); www.lantmateriet.se/sv/Kartor-och-geografisk-information/geodataprodukter/produktlista/markhojdmodell-nedladdning-grid-50/. [Swedish Mapping, Cadastral and Land Registration Authority, Elevation Model download].

74. B. P. Buchanan, M. Fleming, R. L. Schneider, B. K. Richards, J. Archibald, Z. Qiu, M. T. Walter, Evaluating topographic wetness indices across central new york agricultural landscapes. *Hydrol. Earth Syst. Sci.* **18**, 3279–3299 (2014).

75. P. N. Murphy, J. Ogilvie, K. Connor, P. A. Arp, Mapping wetlands: A comparison of two different approaches for New Brunswick, Canada. *Wetlands* **27**, 846–854 (2007).

76. K. J. Beven, M. J. Kirkby, A physically based, variable contributing area model of basin hydrology. *Hydrol. Sci. Bull.* **24**, 43–69 (1979).

77. A. H. Schweiger, S. D. H. Irl, M. J. Steinbauer, J. Dengler, C. Beierkuhnlein, Optimizing sampling approaches along ecological gradients. *Methods Ecol. Evol.* **7**, 463–471 (2016).

78. J. Olsson, J. Södling, F. Wetterhall, Högupplösta nederbördsdata för hydrologisk modellering: En förstudie (2013); www.smhi.se/publikationer/hogupplosta-nederbordsdata-for-hydrologisk-modellering-en-forstudie-1.32746. [High-resolution precipitation data for hydrological modeling: A feasibility study].

79. T. Kristensen, M. Ohlson, P. Bolstad, Z. Nagy, Spatial variability of organic layer thickness and carbon stocks in mature boreal forest stands—Implications and suggestions for sampling designs. *Environ. Monit. Assess.* **187**, 521 (2015).

80. Canadian Agricultural Services Coordinating Committee, *The Canadian System of Soil Classification* (NRC Research Press, 1998).

81. B. G. Jonsson, M. Ekström, P.-A. Esseen, A. Grafström, G. Ståhl, B. Westerlund, Dead wood availability in managed Swedish forests–Policy outcomes and implications for biodiversity. *Forest Ecol. Manag.* **376**, 174–182 (2016).

82. M. B. Bodí, D. A. Martin, V. N. Balfour, C. Santin, S. H. Doerr, P. Pereira, A. Cerda, J. Mataix-Solera, Wildland fire ash: Production, com position and eco-hydro-geomorphic effects. *Earth Sci. Rev.* **130**, 103–127 (2014).

83. K. O. L. Hounkpatin, J. Stendahl, M. Lundblad, E. Karltun, Predicting the spatial distribution of soil organic carbon stock in Swedish forests using a group of covariates and site-specific data. *SOIL* **7**, 377–398 (2021).

84. Swedish University of Agricultural Sciences, Slu carbon data (2023), https://skogsdatalab.direct.quickconnect.to:5001/fsdownload/ZaiJkVbdU/CarbonMaps [accessed 15 February2024].

85. A. M. Ågren, E. M. Hasselquist, J. Stendahl, M. B. Nilsson, S. S. Paul, Delineating the distribution of mineral and peat soils at the landscape scale in northern boreal regions. *SOIL* **8**, 733–749 (2022).

86. J. O'Brien, J. K. Hiers, J. M. Varner, C. M. Hoffman, M. B. Dickinson, S. T. Michaletz, E. L. Loudermilk, B. W. Butler, Advances in mechanistic approaches to quantifying biophysical fire effects. *Curr. Forest. Rep.* **4**, 161–177 (2018).

87. T. H. Deluca, C. Boisvenue, Boreal forest soil carbon: Distribution, function and modelling. *Forestry* **85**, 161–184 (2012).

88. G. Granath, P. A. Moore, M. C. Lukenbach, J. M. Waddington, Mitigating wildfire carbon loss in managed northern peatlands through restoration. *Sci. Rep.* **6**, 28498 (2016).

89. K. Nelson, D. Thompson, C. Hopkinson, R. Petrone, L. Chasmer, Peatland-fire interactions: A review of wildland fire feedbacks and interactions in Canadian boreal peatlands. *Sci. Total Environ.* **769**, 145212 (2021).

90. M. Maljanen, B. D. Sigurdsson, J. Guðmundsson, H. Óskarsson, J. T. Huttunen, P. J. Martikainen, Greenhouse gas balances of managed peatlands in the nordic countries present knowledge and gaps. *Biogeosciences* **7**, 2711–2738 (2010).

91. Statistics Sweden, Population statistics (2024), www.statistikdatabasen.scb.se/pxweb/sv/ssd/START__BE__BE0101/ [accessed 15 February 2024].

92. N. Gillett, A. Weaver, F. Zwiers, M. Flannigan, Detecting the effect of climate change on canadian forest fires. *Geophysical Research Letters* **31**, L18211 (2004).

93. P. Smith, J. F. Soussana, D. Angers, L. Schipper, C. Chenu, D. P. Rasse, N. H. Batjes, F. van Egmond, S. McNeill, M. Kuhnert, C. Arias-Navarro, J. E. Olesen, N. Chirinda, D. Fornara, E. Wollenberg, J. Álvaro-Fuentes, A. Sanz-Cobena, K. Klumpp, How to measure, report and

verify soil carbon change to realize the potential of soil carbon sequestration for atmospheric greenhouse gas removal. *Glob. Change Biol.* **26**, 219–241 (2020).

94. S. Zoltai, L. Morrissey, G. Livingston, W. de Groot, Effects of fires on carbon cycling in north american boreal peatlands. *Environ. Rev.* **6**, 13–24 (1998).

95. B. Buma, K. Hayes, S. Weiss, M. Lucash, Short-interval fires increasing in the alaskan boreal forest as fire self-regulation decays across forest types. *Sci. Rep.* **12**, 4901 (2022).

96. K. Hayes, B. Buma, Effects of short-interval disturbances continue to accumulate, overwhelming variability in local resilience. *Ecosphere* **12**, e03379 (2021).
